# Correction: Multiscale adaptive analysis of circadian rhythms and intradaily variability: Application to actigraphy time series in acute insomnia subjects

**DOI:** 10.1371/journal.pone.0188674

**Published:** 2017-11-20

**Authors:** The PLOS ONE Staff

## Notice of Republication

This article was republished on October 26, 2017, to correct errors in Figs 3, 4, 5, 8, and 9 that were introduced during the typesetting process. The publisher apologizes for the errors. Please download this article again to view the correct version. The originally published, uncorrected article and the republished, corrected articles are provided here for reference.

## Supporting information

S1 File Originally published, uncorrected article. (PDF)

S2 File Republished, corrected article. (PDF)
