# Supplementary material for: Multiscale adaptive analysis of circadian rhythms and intradaily variability: Application to actigraphy time series in acute insomnia subjects
Source: PLoS One. 2017 Jul 28;12(7):e0181762. doi: 10.1371/journal.pone.0181762 (PMC5533453; doi:10.1371/journal.pone.0181762)
Supplement: S1 File — Calculations with Fourier spectral analysis, cosinor, intradaily variability (IV), singular spectrum analysis (SSA), kurtosis and linear trend, and detrended fluctuation analysis (DFA). (PDF) [file pone.0181762.s001.pdf]

ONLINE SUPPORTING INFORMATION:

Multiscale adaptive analysis of circadian rhythms and intradaily variability: Application to actigraphy time series in acute insomnia subjects

Ruben Fossion, Ana Leonor Rivera, Juan C. Toledo-Roy, Jason Ellis and Maia Angelova

I. FOURIER SPECTRAL ANALYSIS

The traditional technique to study time series is Fourier spectral analysis which decomposes a discrete time series $x(n) = x_1, x_2, \ldots x_N$ of N successive observations as the sum of periodic sine and cosine functions,

$$\begin{aligned} f(t) &= \sum_{f=0}^{N/2} A_k \sin(2\pi f t + \phi_k) \\ &= \sum_{f=0}^{N/2} (a_k \cos(2\pi f t) + b_k \sin(2\pi f t)) \\ &= \sum_{f=-N}^{N} c_k e^{i\omega_k t} \end{aligned} \tag{1}$$

where the phase ϕ_k of each component is considered either explicitely, or as the weighted sum of a sine and a cosine function with the same frequency, or as a complex sum over positive and negative frequencies. According to the Parseval theorem, each periodic function contributes with a *partial variance* $P(f)$, such that the sum of the partial variances of all time-series components is equal to the total variance Var of the time series,

$$\text{Var} \equiv 2 \sum_{f=1}^{N/2} P(f), \tag{2}$$

where we take into account that the constant DC term with $f = 0$ does not carry any variance, and the components with the same frequency but different sign carry the same amount of partial variance.

The population-average of the Fourier power spectra for the asymptomatic controls and the acute insomnia subjects are very similar, see Fig. 1 (left-hand panel). A first feature of these power spectra is the dominant peak at the characteristic frequency $f = 1/24$h, which is the reflection in the frequency domain of the periodic circadian cycle of 24h of Fig. 4, and successive smaller peaks at multiples of this frequency which are the higher harmonics 2/24h, 4/24h, etc., and it can be observed that the controls and acute insomnia subjects have circadian cycles of comparable magnitude. A second feature is the power-law tail $P(f) \propto 1/f^{\beta}$ with $\beta \approx 1$ in the frequency range $1.5 \le \log_{10} f \le 3.5$ (1/5hrs$\le f \le$1/3min), which is typical for fractal physiological processes such as heart rate variability and indicates scale invariance and the absence of a characteristic period. One difference between the asymptomatic controls and the acute insomnia subjects appears to be a higher variability in a limited frequency range around $f = 1/90$min, but a Kruskal-Wallis test failed to indicate a significant difference between the Fourier power spectra of the two populations.

A difficulty in the comparison of power spectra is the large dispersion at high frequencies. An alternative way to represent the power spectrum is to reorder the partial variances $P(f)$ not as a function of frequency $f = 0, \ldots, N/2$, but ordered according to magnitude $P(k)$ from the largest partial variance to the smallest, for $k = 1, \ldots, N$, where it is customary to present the components corresponding to both the postitive and the negative frequencies. Such a visual representation is called *Zipf plot* or *scree diagram*, and eliminates the large dispersion, while conserving power laws present in the spectrum, $P(k) \propto 1/k^{\gamma}$ with $\gamma \approx 1$, for the whole range $0.5 \le \log_{10} k \le 3.5$ in the case of the asymptomatic controls, whereas in the case of the acute insomnia subjects this scale invariance appears to be broken with a crossover between a somewhat shallower slope below $\log_{10} k \lesssim 2.2$ and a somewhat steeper slope above $\log_{10} k \gtrsim 2.2$. This is reminiscent of what happens in heart rate variability, where the typical $1/f$ noise scale invariance of healthy controls is broken in the case of ageing [3] and congestive heart failure [4]. In the present

case, using Fourier spectral analysis, the difference between asymptomatic controls and acute insomnia subjects at ultradian scales does not reach statistical significance.

There is a very large similarity between the scree diagram as calculated here from Fourier spectral analysis in Fig. 1 (right-hand panel) and the scree diagram calculated from SSA in Fig. 9 of the main manuscript. Fourier spectral analysis makes the assumption that a discrete time series $x(n) = x_1, x_2, \ldots, x_N$ of length N can be decomposed as the superposition of $f_{\max} = N/2$ independent oscillators, where $f_{\max}$ is the maximum frequency as given by the Nyquist theorem. As discussed in the main manuscript, SSA considers the parameter L, which allows to control the number of time-series components $g_k(n)$ with $k = 1, \ldots, r$ in which to decompose the time series $x(n)$, where $r \leq \mathrm{Min}[K, L]$ with $K = N - L + 1$. In the limit for $L \to N/2$, it can be shown that SSA converges towards Fourier [5]; for smaller values of the main parameter of SSA analysis, $L < N/2$, several Fourier components are compressed in one single SSA component, allowing the very economic description of quasi-periodic modes for which Fourier otherwise would need a superposition of many sine and cosine functions. Another important difference is that the SSA time-series components $g_k(n)$ usually are non-stationary. In particular, it has been noted that the 90min ultradian waking BRAC cycles are more present in the mornings and tend to be "masked" in the evenings, and that if 24h time series are analyzed on the whole using a stationary method such as Fourier, then results for the 90min cycle will be strongly attenuated [19]. This might be the reason why using Fourier no significant differences are obtained between the two populations, whereas SSA does indicate a significant increase in ultradian variability near $\langle f \rangle = 1/90\text{min}$ for the acute insomnia subjects.

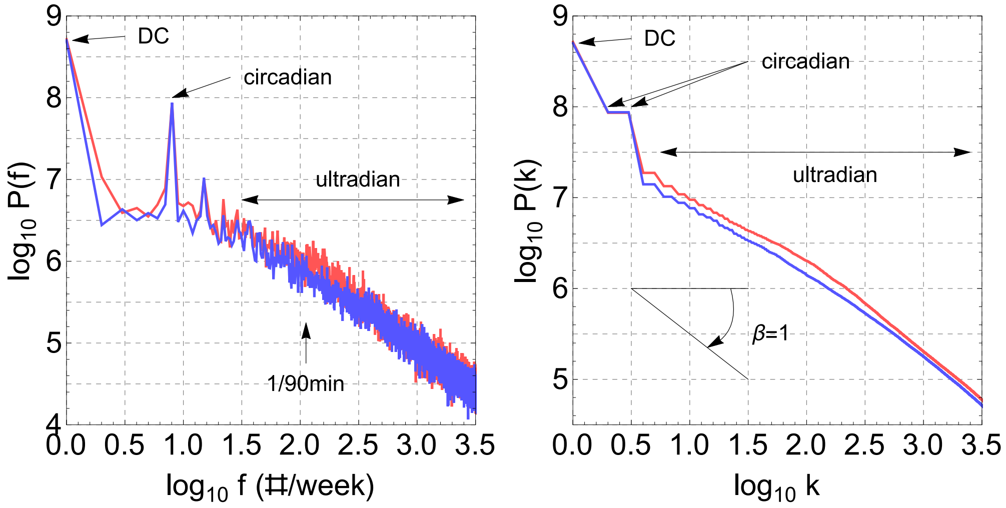

FIG. 1: **Fourier spectral analysis of weekly actigraphy time series.** Power spectra shown with positive frequency components ordered according to frequency, with positions of the specific frequencies of 1/day and 1/hour indicated by arrows (left-hand panel) and with components ordered according to partial variance (right-hand panel).

II. COSINOR

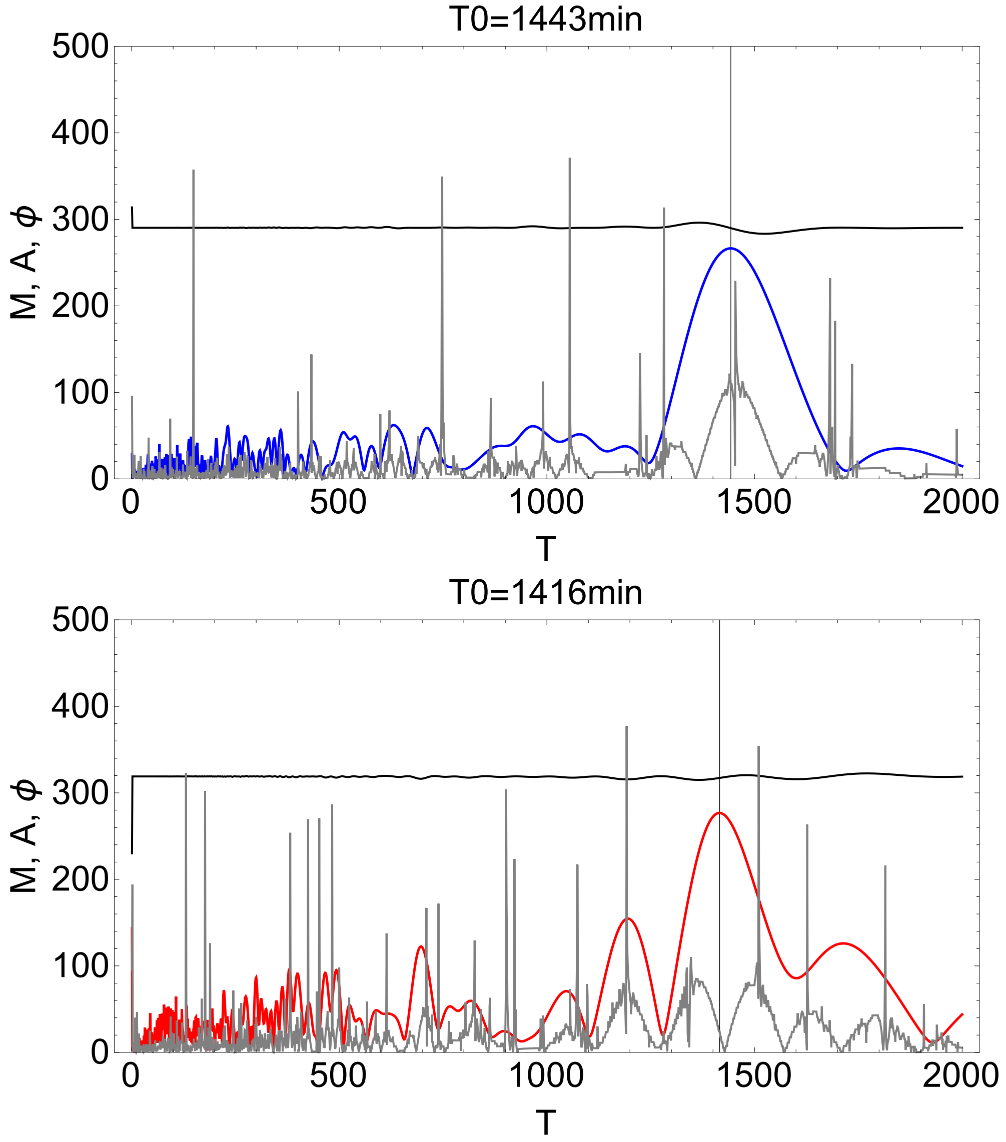

FIG. 2: **Determination of period T in cosinor analysis for individual subjects.** Shown for the control (upper panel) and the acute insomnia subject (bottom panel) of the time series of Fig.1 of the main article. The cosinor model was fitted to the 1-week actigraphy time series for successive values of period T in the range $1 \leq T \leq 2000$min and for each specific value of T the other parameters of mesor M (black curve), amplitude A (blue or red curve) and phase ϕ (grey curve) were determined by least-square fitting. Period T was determined as the value that maximizes amplitude A (shown as T_0 on top of each graph, and as vertical axis).

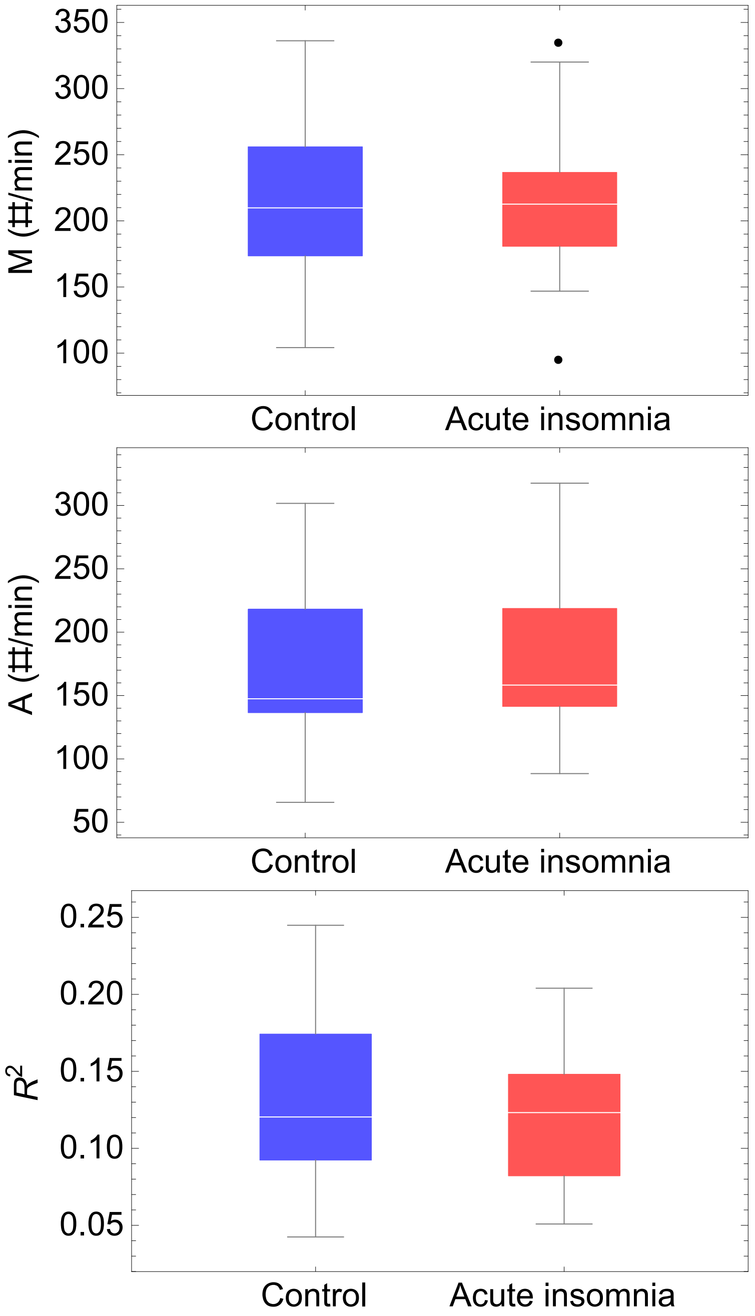

FIG. 3: **Results for circadian parameters of cosinor analysis**. Shown are box-and-whisker plots for mesor M, amplitude A and coefficient of determination R^2 for controls (blue boxes) and acute insomnia subjects (red boxes).

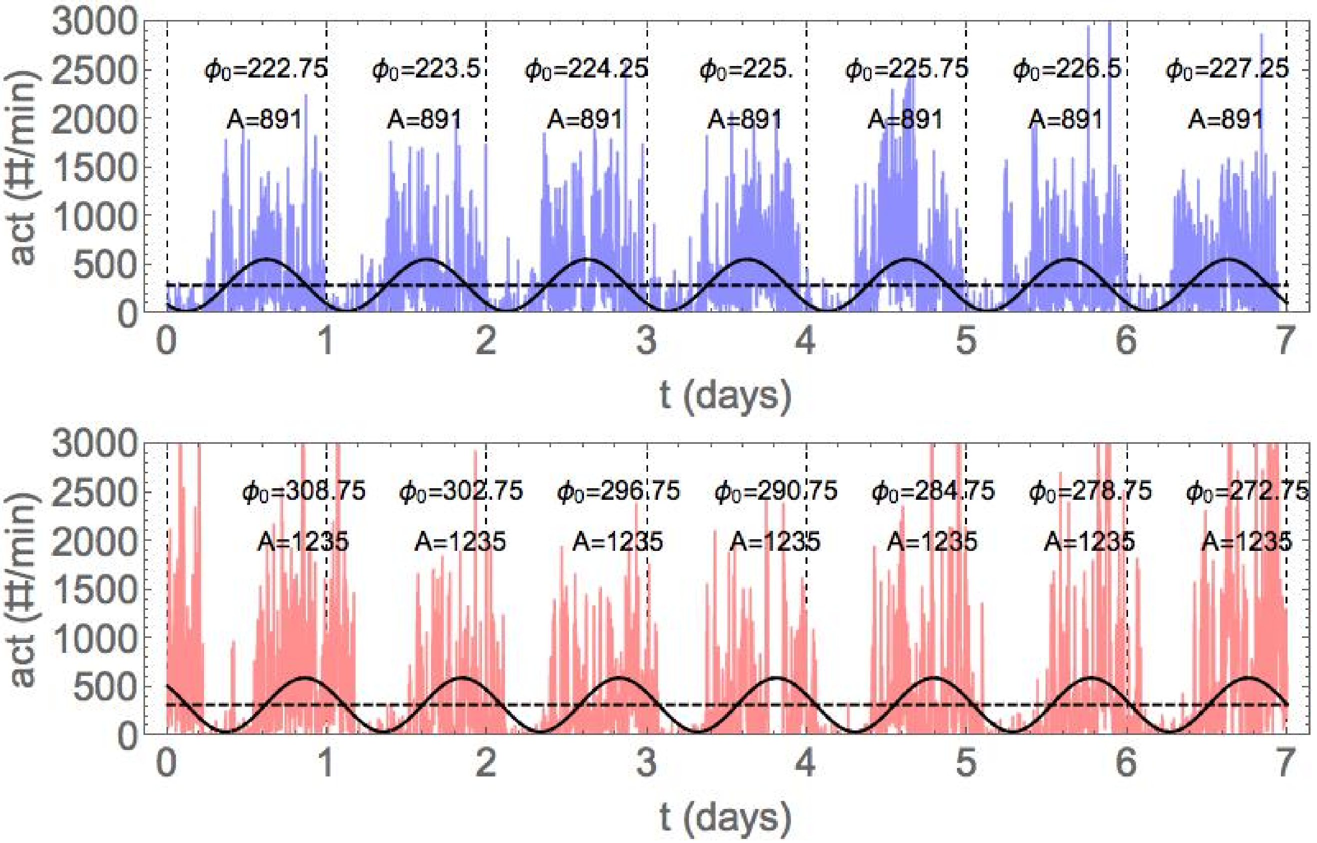

FIG. 4: **Mesor and circadian component according to cosinor analysis**. Shown for the 1-week actigraphy series of Fig. 1 of the main article, for the control subject with mesor $M = 290$, amplitude $A = 266$, 7-day average acrophase $\phi_0 = 225°$ and $R^2 = 0.223$ (upper panel) and the acute insomnia subject with $M = 319$, $A = 267$, 7-day average $\phi_0 = 291°$ and $R^2 = 0.124$ (bottom panel). Shown are the constant mesor value M (dashed line), the cosinor fit of the circadian cycle (full curve), and the day-to-day values for amplitude A (constant) and acrophase ϕ_0 (varying). Vertical gridlines at midnight at 24h intervals.

III. INTRADAILY VARIABILITY

A time series can be considered to consist of a trend, dominant periodic or quasi-periodic oscillations and superposed irregular fluctuations. Often, a dominant trend or dominant oscillations make it impossible to study statistics of the superposed fluctuations. The technique of intradaily variability applies the simple procedure of considering the derivative of the time series (differences of successive times-series values), which cancels out the trend and circadian oscillation, given that these latter time-series components can be considered to be slow with respect to the sampling frequency P. If the sampling interval is not very small compared to the time scale of the trend or dominant oscillations, than part of these will still be present in the difference series. Examples of time-series derivatives and resampling are shown for a control in Fig. 5 and for an acute insomnia subject in Fig. 6.

Results for intradaily variability $\mathrm{IV}(P)$ as a function of the sampling interval P are shown in Fig. 7 for different normalization factors. If no normalization is applied (upper panel), then one studies only the variance of the difference series at different scales P. In the case of the acute insomnia subjecs, two relative maxima can be observed, a first one near sample interval $P = 20$min, and a second one near sample interval $P = 500$min. The first relative maximum is due to high-frequency fluctuations, whereas the second one is due to the circadian cycle. In the case of the asymptomatic controls, the second relative maximum near $P = 500$min is also present, with a similar magnitude, but the first relative maximum near $P = 20$min is absent. This suggests that the difference between the two populations is due to a larger intradaily variability of the subjects with acute insomnia. Note that the difference between both populations is largest not at the traditional scale of $P = 60$min, as orgininaly proposed in Ref. [1], but for smaller scales $10 \leq P \leq 50$min. On the other hand, it is possible that differences between two populations are due to the global variance of the whole time series, and not to the variability at a certain scale. Therefore, traditionally, the intradaily variability values are normalized. In Ref. [2], the variance of the difference series at specific scale P, $\mathrm{Var}(X'_P)$, was scaled by the variance of the corresponding series at that scale P, $\mathrm{Var}(X_P)$. However, in this case $\mathrm{IV}(P)$ becomes a composed function and we can appreciate that the behaviour of this function is different from the original non-scaled function (bottom panel). Therefore, in this work we proposed to rescale the function $\mathrm{IV}(P)$ with the variance of the original time series, $\mathrm{Var}(x)$, such that $IV(P)$ is a simple function with the same behaviour as the non-scaled function $\mathrm{IV}(P)$. We can appreciate that the acute insomnia subjects have a slightly smaller variability at the scale of the circadian cycle, but a larger intradaily variability in the range $10 \leq P \leq 50$min. The differences however are not statistically significant.

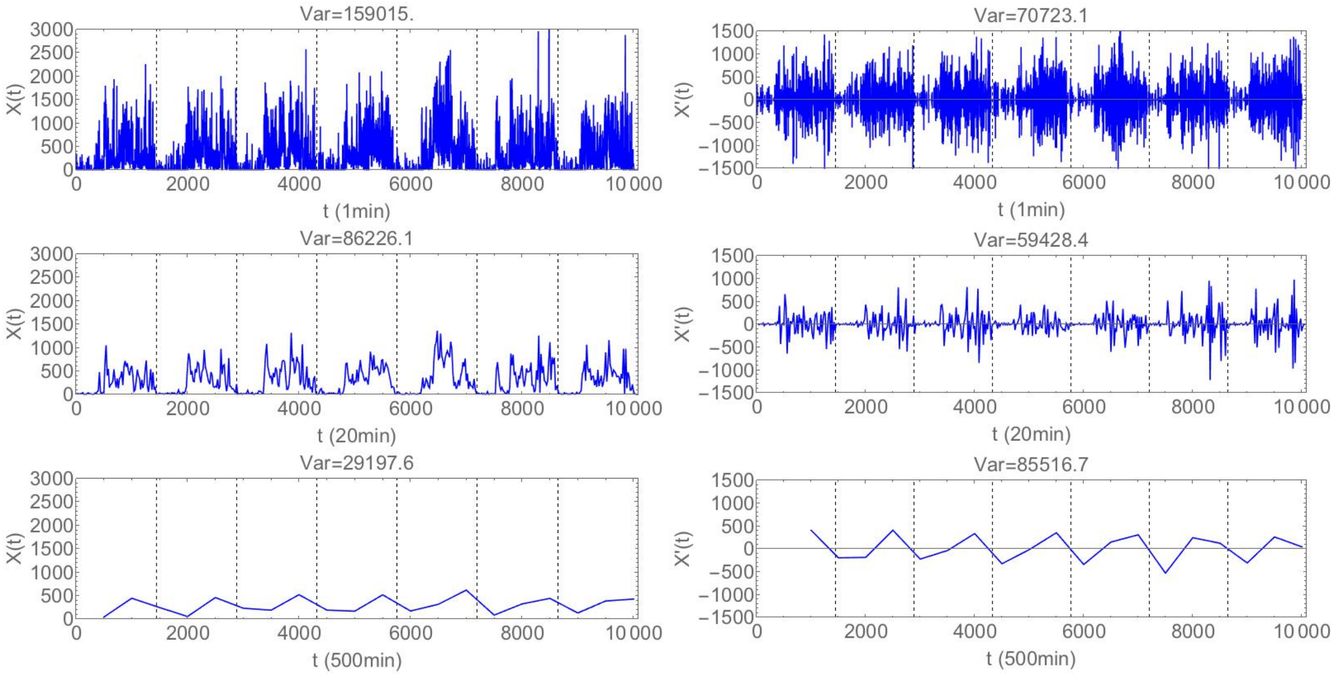

FIG. 5: **Variance of actigraphy series and difference series as a function of sampling interval P.** Shown are the actigraphy time series $X_P(n)$ of the particular control subject of Fig. 1 of the main manuscript for different sample intervals $P = 1$min, $P = 20$min and $P = 500$min (left-hand panels), and the corresponding difference series $X'_P(n) = X_P(n) - X_P(n-1)$ (right-hand panels).

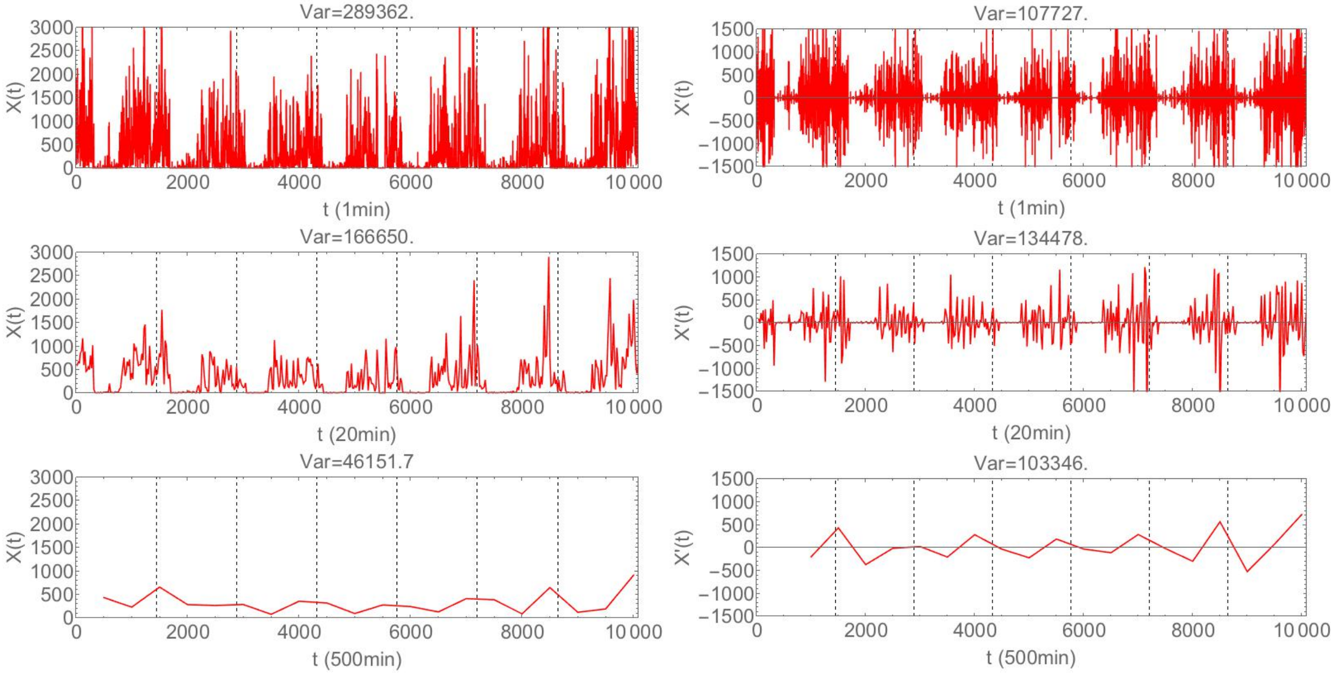

FIG. 6: **Variance of actigraphy series and difference series as a function of sampling interval** P**.** Shown is the actigraphy time series $X_P(n)$ of the particular acute insomnia subject of Fig. 1 of the main manuscript for different sample intervals $P = 1$min, $P = 20$min and $P = 500$min (left-hand panels), and the corresponding difference series $X'_P(n) = X_P(n) - X_P(n-1)$ (right-hand panels).

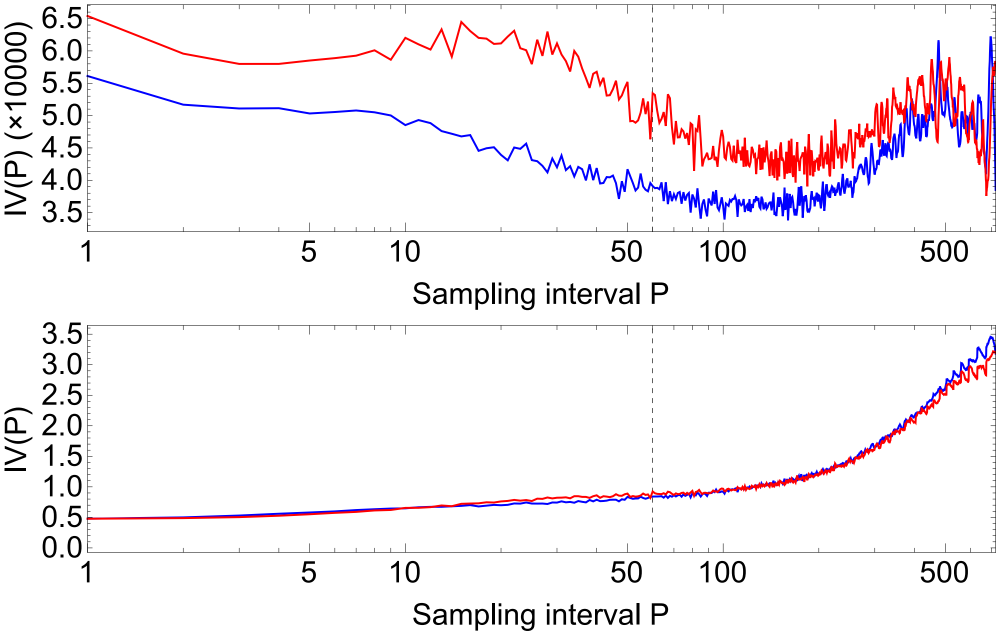

FIG. 7: **Intradaily variability** $\mathrm{IV}(P)$ **as a function of sample interval**. Shown without normalization $\mathrm{IV}(P) = \mathrm{Var}(X'_P)$ (upper panel), and normalized with the variance of the resampled time series $\mathrm{IV}(P) = \mathrm{Var}(X'_P)/\mathrm{Var}(X_P)$ (bottom panel). Shown for the population of asymptomatic controls (blue) and acute insomnia subjects (red). Vertical gridline at traditional sample interval $P = 60$min.

IV. SSA

As discussed in the Methodology section on SSA in the main manuscript, time-series components $g_k(n)$ with $k = 1, \ldots, r$ are not necessarily uncorrelated. Two visual tools to estimate the degree of uncorrelatedness of time-series components are the scree diagram and the w-correlation matrix [7–9]. In Fig. 8 and 9, the decomposition of two actigraphy time series is demonstrated. Only a few selected time-series components are plotted: $g_1(n), g_2(n), g_3(n), g_4(n), g_5(n), g_{30}(n), g_{40}(n)$.

The scree diagram shows the partial variance λ_k carried by each of the time-series components $g_k(n)$. Time-series components $g_k(n)$ and $g_l(n)$ that have similar partial variances $\lambda_k \approx \lambda_l$ are likely to be correlated. E.g., in the present study, components $g_2(n)$ and $g_3(n)$ together describe the circadian rhythm, it can be observed that both oscillate with the same average frequency $\langle f \rangle = 1/1440\text{min}$, within the Fourier approach they would correspond to the sine and cosine with the same frequency. On the other hand, time-series components that have partial variances λ_k that are far apart are very likely to be uncorrelated, in this way, 3 major mutually uncorrelated contributions can be distinguished: the dominant non-oscillating trend component $g_1(n)$, the circadian rhythm $g_2(n) + g_3(n)$ and higher-order ultradian fluctuations $g_k(n)$ for $k \geq 4$.

The w-correlation matrix shows graphically the w-weighted Pearson correlation coefficient $r_{k,l}$ between each pair of time-series components $g_k(n)$ and $g_l(n)$. The w-correlation matrix confirms the conclusions drawn from the scree diagram that the trend component $g_1(n)$ and the circadian rhythm $g_2(n) + g_3(n)$ can be considered independently from the higher-order ultradian components $g_k(n)$ for $k \geq 4$.

The average frequency $\langle f \rangle$ of the time-series components can be estimated by dividing the length of the time series by the number of oscillations, or equivalently, by applying Fourier spectral analysis and defining the frequency of its most dominant peak. In this way, it can be demonstrated that the average frequency of the range of scales with increased ultradian variability for the acute insomnia subjects near $k = 30$ (or $\log_{10}(k) = 1.5$) is in the $\langle f \rangle = 1/90 - 1/60\text{min}$ range.

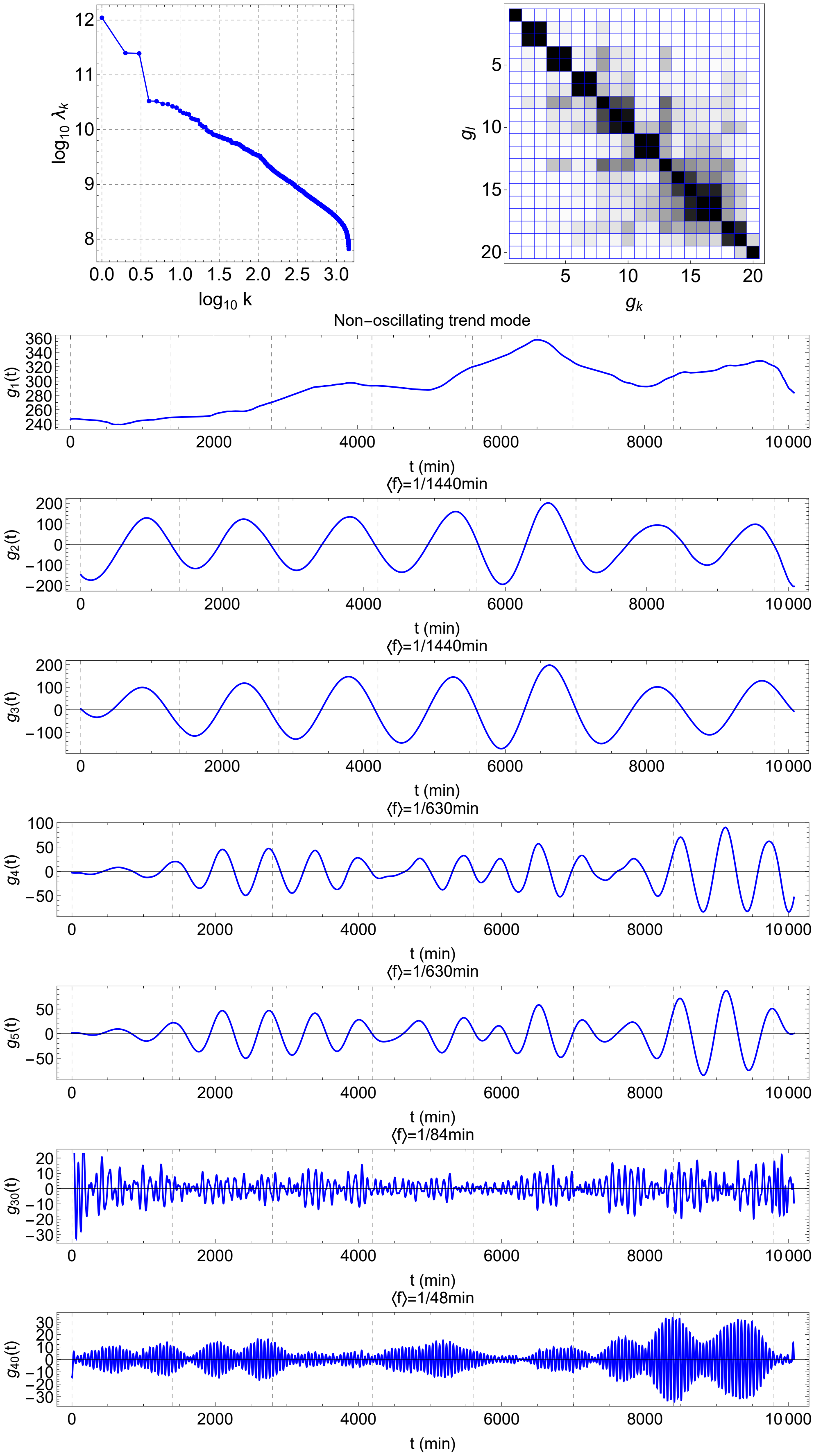

FIG. 8: **SSA time-series decomposition**. Scree diagram, $w-$correlation matrix and some of the resulting components $g_k(n)$ with $k = 1 - 5, 30, 40$. Applied to the actigraphy time series of the control subject of Fig. 1 of the main manuscript.

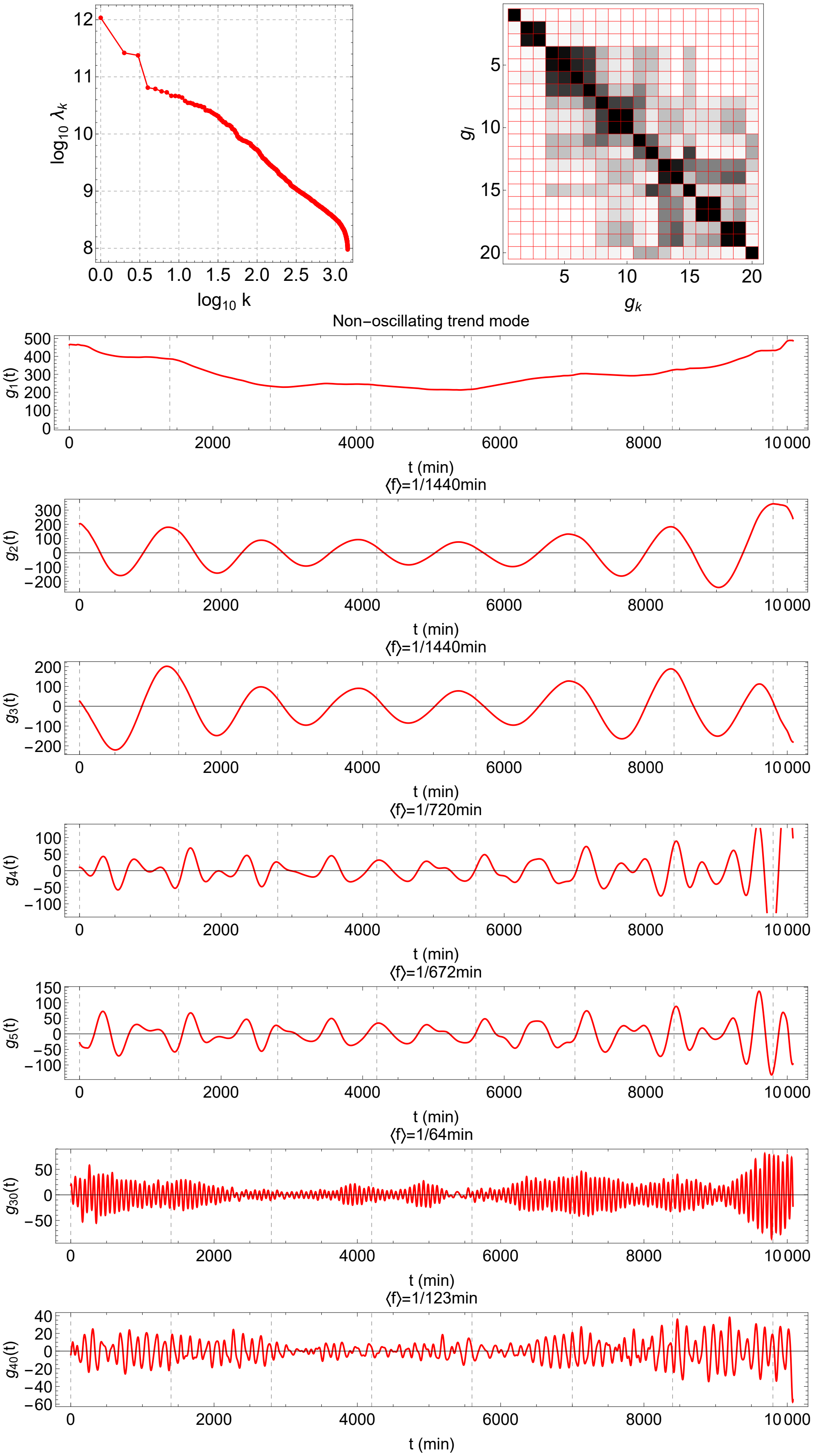

FIG. 9: **SSA time-series decomposition**. Scree diagram, $w-$correlation matrix and some of the resulting components $g_k(n)$ with $k = 1-5, 30, 40$. Applied to the actigraphy time series of the subject with acute insomnia of Fig. 1 of the main manuscript.

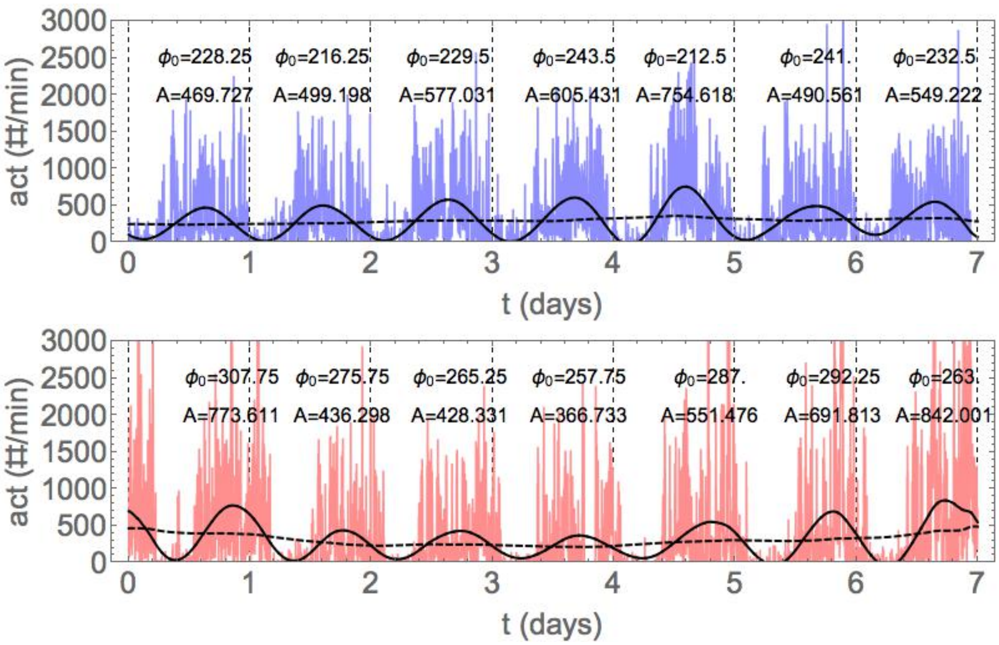

FIG. 10: **Circadian component according to SSA analysis**. Shown for the 1-week actigraphy series of Fig. 1 of the main article, for the control subject with 7-day average values for mesor $M = 297$, amplitude $A = 584$ and acrophase $\phi_0 = 278$ (upper panel) and the subject with acute insomnia subject with 7-day average values $M = 307$, $A = 584$ and $\phi_0 = 278$ (bottom panel). Shown are the time-varying mesor trend $g_1(n)$ (dashed curve), the time-varying circadian cycle $g_2(n) + g_3(n)$ (full curve), and the day-to-day varying values for amplitude A and acrophase ϕ_0. Vertical gridlines at 24h intervals at midnight.

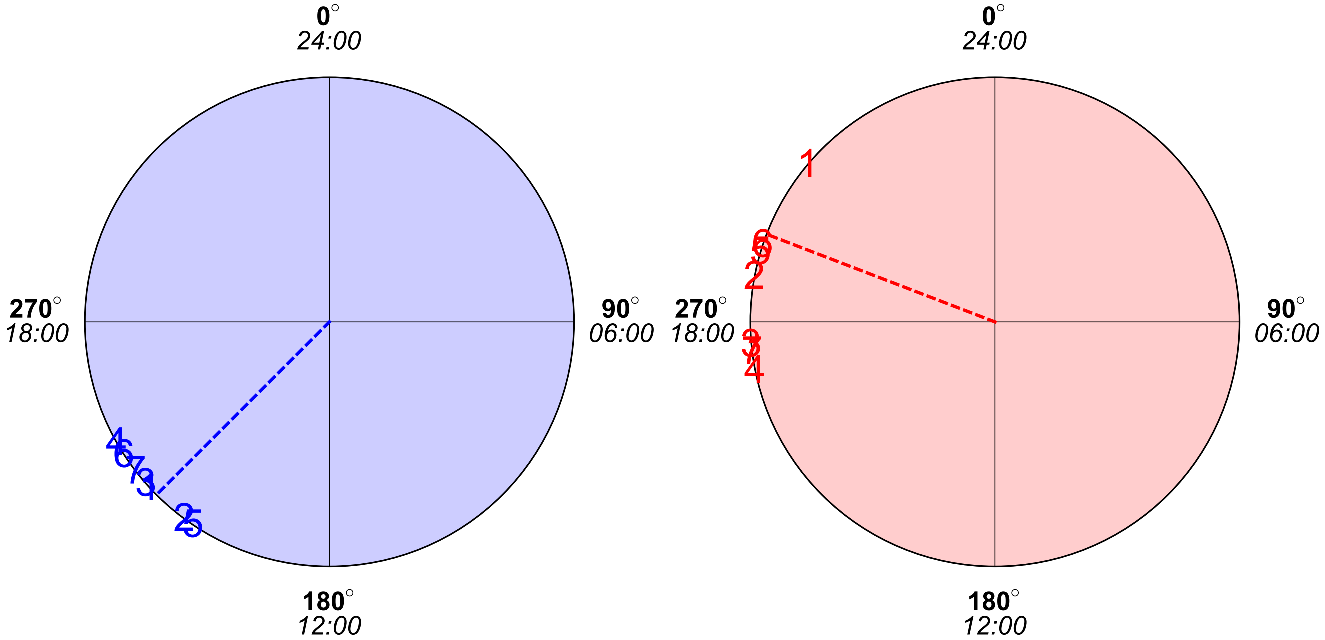

FIG. 11: **Acrophase ϕ_0 dial plots**. Shown for the specific control subject (left-hand panel) and the specific acute insomnia subject (right-hand panel) of Fig.1 of the main manuscript. Shown are the cosinor week-average estimate (dashed line) and the day-per-day values as determined by SSA analysis (numbers indicating the successive days).

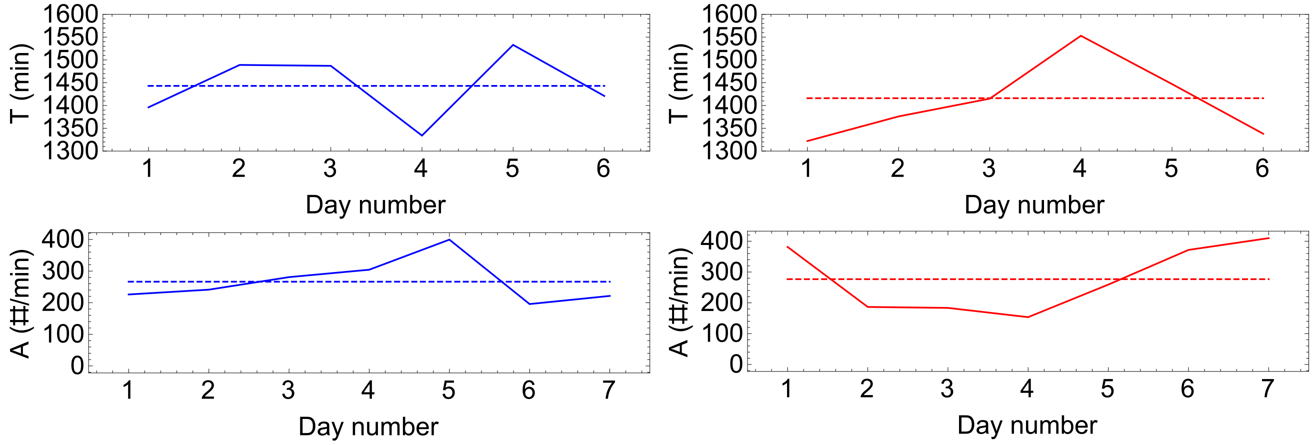

FIG. 12: **Variability of circadian parameters.** Cosinor vs. SSA. Parameters of the circadian cycle are constant or vary linearly in the cosinor model (dashed lines). SSA (full curves) is capable of describing day-to-day variation of the parameter values in the circadian cycle. Shown for the specific control subject (left-hand panels) and for the specific acute insomnia subject (right-hand panels) of the previous figures.

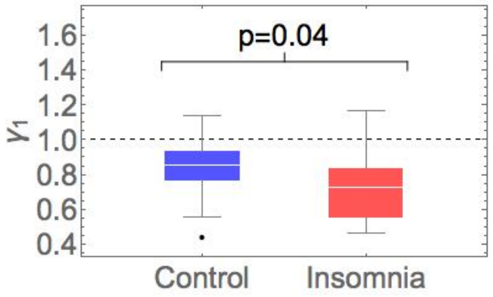

(a) Large time scales (> 90min BRAC scales)

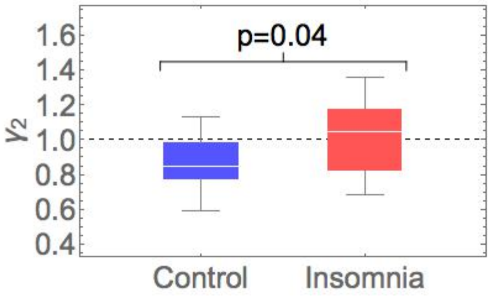

(b) Small scales ($\lesssim$ 90min BRAC scales)

FIG. 13: **Fractal scaling according to SSA analysis**. Box-whisker plots of the scaling exponent of the power law $\lambda_k \propto 1/k^\gamma$ for the control subjects (blue) and the acute insomnia patients (red) in 2 different scaling regions, (a) scaling exponent γ_1 in the larger-scale region $0.8 \leq \log_{10} k \leq 1.5$ and (b) scaling exponent γ_2 and in the smaller scale region $1.6 \leq \log_{10} k \leq 2$.

V. KURTOSIS AND LINEAR TREND

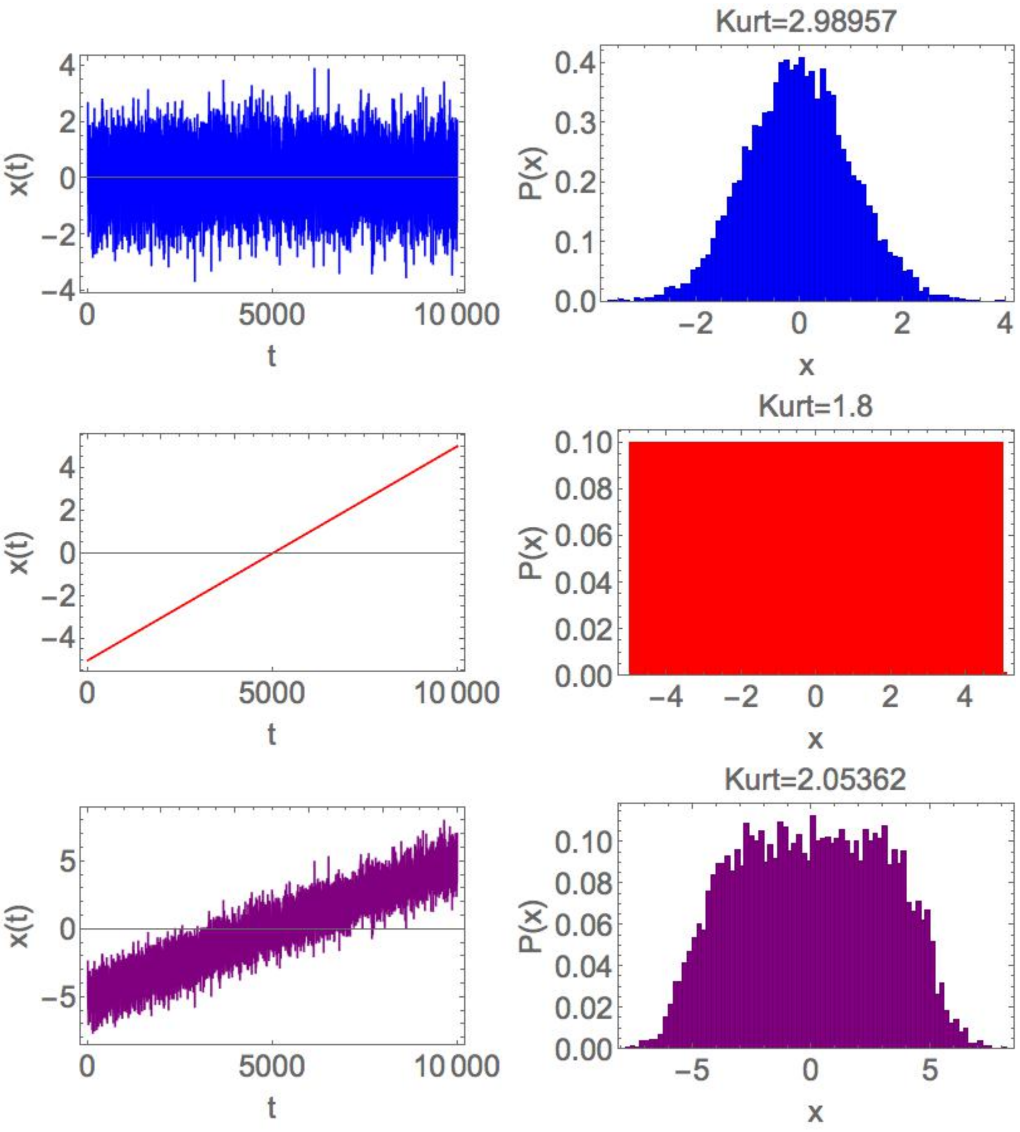

FIG. 14: **Relation between kurtosis and linear trend**. Shown are some time series (left-hand panels) and corresponding distributions (right-hand panels). Gaussian distributed fluctuations (Kurt=3) around a constant value (upper row), a linear trend and a corresponding uniform distribution (second row), Gaussian distributed fluctuations around a linear trend resulting in platykurtic (Kurt<3) distribution (bottom row). can result in a platycurtic distribution.

VI. DETRENDED FLUCTUATION ANALYSIS (DFA)

Detrended fluctuation analysis (DFA) is applied to time series to calculate fractal scaling exponents with more precision than, e.g., Fourier spectral analysis. However, one needs to be careful with its application to time series with trends [10–12] or periodicities [13, 14] as these can produce artefacts. DFA is not calculated directly on the original time series $x(t)$, but on the integrated time series,

$$y(\tau) = \sum_{t=1}^{\tau} (x(t) - \langle x \rangle) \tag{3}$$

where $\langle x \rangle$ is the average value of $x(t)$. This integrated time series $y(\tau)$ is divided into boxes of equal length n (in units of minutes), and each box is detrended by subtracting a least-squares fit, denoted by $y_n(\tau)$. The fluctuation function,

$$F(n)) = \sqrt{\frac{1}{T} \sum_{\tau=1}^{T} \Big(y(\tau) - y_n(\tau) \Big)^2}, \tag{4}$$

is calculated for all boxes of each size n, and this process is repeated for a range of box sizes n to calculate the relationship between the mean fluctuation $F(n)$ as a funtion of the box size. For fractal time series, the fluctuation function behave as a power law $F(n) \propto n^{\alpha}$, where α is the scaling exponent.

Actigraphy time series over multiple days are characterized by a dominant circadian component with much smaller superposed fluctuations. Therefore, to avoid the quasi-periodicity of the circadian cycle, DFA must be applied to day-time fragments [15–17] or night-time fragments [18] separately, and a $1/f$ scaling behaviour can be found. The purpose of the present section is to a DFA analysis of day-time fragments with the results on fractal scaling of the main manuscript where SSA was applied to 1-week continuous actigraphy time series. Taking in mind that BRAC cycles are observed especially during morning hours while they tend to disappear towards the evening [19], in the following, we will focus on day-time fragments from 10:00 till 16:00 with a total length of $T = 360$min and we investigate the scaling properties for windows in the range of $n = 4 - 180$min. Examples of day-time fragments $x(t)$ are shown in Fig. 15 for a control subject (panel (a)) and a subject with acute insomnia (panel (b)), where also the corresponding integrated time series $y(\tau)$ are presented. In panel (c), the fluctuation function $F(n)$ is shown for the control group and the acute insomnia group, with a scaling exponent $\alpha \approx 1$ for both groups, but being slightly larger for the insomnia group than for the control group. For small scales, below the proposed BRAC scales of $n = 60 - 90$min (shaded intervals), the fluctuation function $F(n)$ is smaller for the insomnia group than for the control group, whereas for larger scales $F(n)$ becomes larger for the insomnia group than for the control group. For each day-time fragment, the scaling exponent α was estimated, and then for each subject the 7-day average exponent $\langle \alpha \rangle$ was calculated. In panels (d-e), group-average exponents $\langle \alpha \rangle$ are compared: for the control group $\langle \alpha_C \rangle = 0.95 \pm 0.07$, for the acute insomnia group $\langle \alpha_I \rangle = 0.99 \pm 0.07$, and a Kruskal-Wallis test resulted in $p = 0.02$, which indicates a small but significant difference between both groups. When other day-time fragments T are considered, with different lengths or at different times of the day, DFA results are similar with $\langle \alpha_C \rangle \approx 0.95$ and $\langle \alpha_I \rangle = 0.99$, but the statistical significance of the difference between both groups tends to decrease towards the evening hours.

When comparing Figs. 13(b) and 15(d) of the Supporting Information, it can be seen that the results on fractal scaling as obtained with DFA and with SSA are similar. DFA is applied to day-time fragments of the time series and thus only probes smaller scales ($n \lesssim$ 60-90min BRAC scales), whereas SSA is applied to many-day continuous time series such that both smaller-scale ($n \lesssim$ 60-90min BRAC scales) and larger-scale ($n >$ 60-90min BRAC scales) aspects of fractal scaling can be quantified. With respect to the smaller-scale aspects as presented in Figs. 13(b) and 15(d), DFA and SSA agree that the scaling exponents, α and γ, respectively, are larger for the insomnia group than for the control group. SSA gives the additional information that at larger scales the scaling exponent γ is smaller for the insomnia group than for the control group, resulting in a crossover behaviour for subjects with acute insomnia whereas the control subjects appear to follow a single power law.

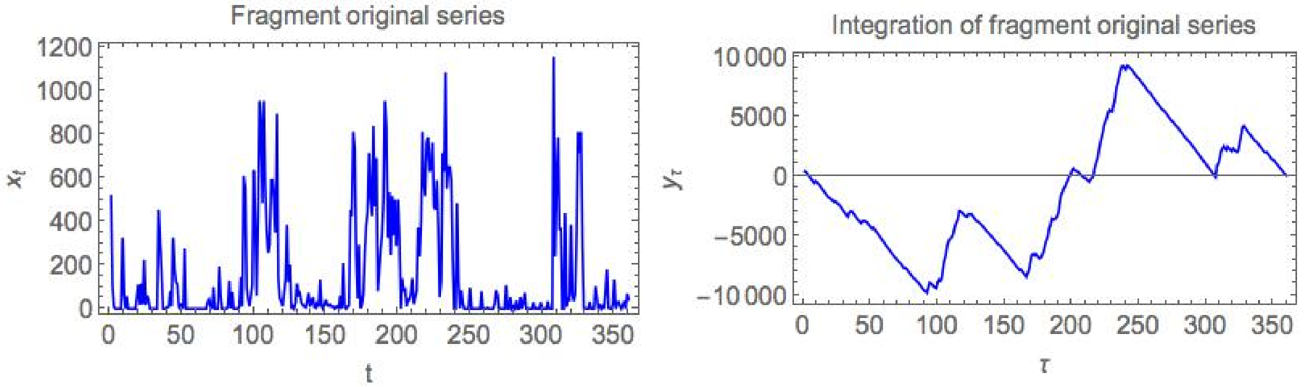

(a) Day-time fragment of control subject and integrated series

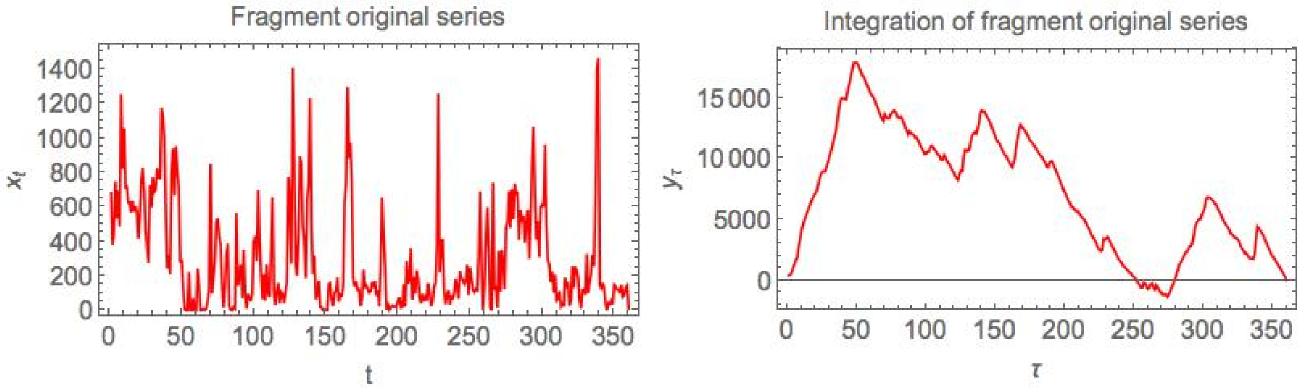

(b) Day-time fragment of acute insomnia subject and integrated series

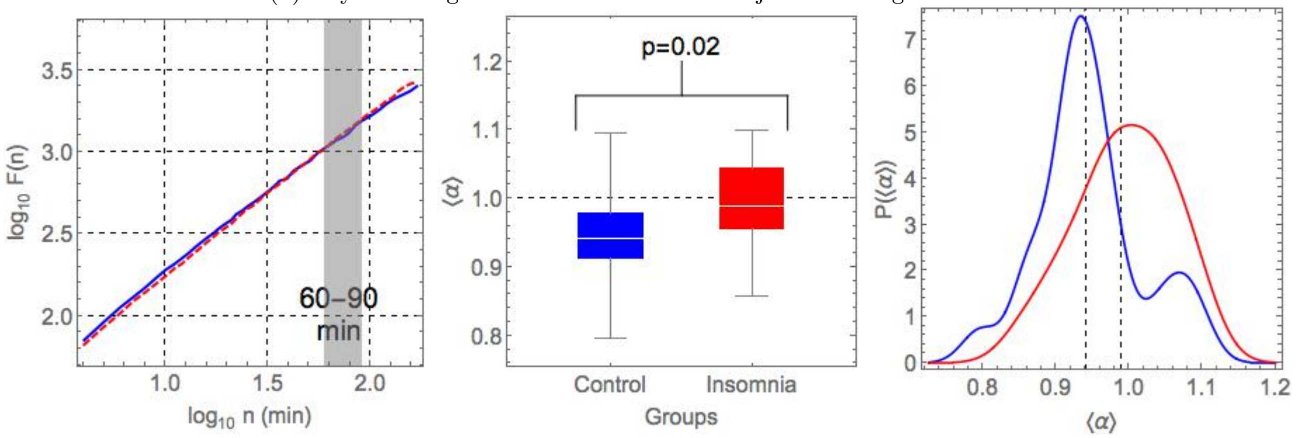

(c) Fluct. func. ($\lesssim$ 90min scales) (d) Box-whisker ($\lesssim$ 90min scales) (e) Distribution

FIG. 15: **DFA analysis.** 6-hour day-time fragment of actigraphy time series and the corresponding integrated time series of (a) a control subject, (b) a subject with acute insomnia, (c) group-averaged fluctuation function for the control subjects (blue continuous curve) and for the subjects with acute insomnia (red dashed curve) where the BRAC time scales of 60-90min are indicated (grey shaded interval), also shown are presentations of the 7-day average scaling exponent $\langle\alpha\rangle$ for control subjects (blue) and for subjects with acute insomnia (red) as (d) a box-whisker plot and (e) a probability density function.

VII. REFERENCES

---

[1] Witting W, Kwa IH, Eikelenboom P, Mirmiran M, Swaab DF Alterations in the circadian rest-activity rhythm in aging and Alzheimer's disease Biol Psych. 1990; 27: 563–572.

[2] Goncalves BSB, Adamowicz T, Mazzilli Louzada F, Moreno CR, Fontenele Araujo J Nonparametric methods in actigraphy: An update Sleep Sci 2014 Sept; 158–164.

[3] Iyengar N, Peng C-K, Morin R, Goldberger AL, Lipsitz LA Age-related alterations in the fractal scaling of cardiac interbeat interval dynamics Am J Physiol 1996; 271: R1078–R1084.

[4] Peng C-K, Havlin S, Hausdorff M, Mietus JE, Stanley HE, Goldberger AL Fractal mechanisms and heart rate dynamics J Electrocardiol 1995; 28: 59–65.

[5] Ghil M, Allen MR, Dettinger MD, Ide K, Kondrashov D, Mann ME, et al. Advanced spectral methods for climatic time series Rev Geophys 2002; 40: 1.

[6] Lavie P. Ultradian rhythms in arousal - The problem of masking. Chronobiol Int. 1989; 6(1): 21-28.

[7] Golyandina N, Nekrutkin V, Zhigljavsky AA Analysis of time series structure: SSA and related techniques Chapman & Hall/CRC: Boca Raton, London, New York, Washington D.C.

[8] Golyandina N, Zhigljavsky A Singular spectrum analysis for time series SpringerBriefs in statistics: Heidelberg, New York, Dordrecht, London

[9] Hassani H Singular Spectrum Analysis: Methodology and comparisons J Data Sci 2007; 5: 239–257.

[10] Hu K, Ivanov P Ch, Chen Z, Carpena P, Stanley H E Effect of trends on detrended fluctuation analysis Phys Rev E 2001; 64: 011114.

[11] Nagarajan R, Kavassen R G Minimizing the effect of trends on detrended fluctuation analysis of long-range correlated noise Phys A 2005; 354: 182–198.

[12] Bryce R M, Sprague K B Revisiting detrended fluctuation analysis Sci Rep 2012; 2: 1–6.

[13] Nagarajan R, Kavasseri R G Minimizing the effect of periodic and quasi-periodic trends in detrended fluctuation analysis Chaos, Solitons and Fractals 2005; 26: 777–784.

[14] Perakakis P, Taylor M, Martinez-Nieto E, Revithi I, Vila J Breathing frequency bias in fractal analysis of heart rate variability Biol Psychol 2009; 82:82–88.

[15] Hu K, Ivanov PCh, Chen Z, Hilton MF, Stanley HE, Shea SA. Non-random fluctuations and multi-scale dynamics regulation of human activity. P Natl Acad Sci USA. 2009; 106(8): 2490-2494.

[16] Ivanov PCh, Hu K, Hilton MF, Shea SA, Stanley HE. Endogenous circadian rhythm in human motor activity uncoupled from circadian influences on cardiac dynamics. P Natl Acad Sci USA. 2007; 104(52): 20702-20707.

[17] Hu K, Van Someren EJW, Shea SA, Scheer FAJL. Reduction of scale invariance of activity fluctuations with aging and Alzheimers disease: Involvement of the circadian pacemaker. P Natl Acad Sci USA. 2009; 106(8): 2490-2494.

[18] Holloway PM, Angelova M, Lombardo S, St Clair Gibson A, Lee David, Ellis J. Complexity analysis of sleep and alterations with insomnia based on non-invasive techniques. Interface 2014 Feb; 11: 20131112.

[19] Lavie P. Ultradian rhythms in arousal - The problem of masking. Chronobiol Int. 1989; 6(1): 21-28.
