# Supplementary material for: Correction: Multiscale adaptive analysis of circadian rhythms and intradaily variability: Application to actigraphy time series in acute insomnia subjects
Source: PLoS One. 2017 Nov 20;12(11):e0188674. doi: 10.1371/journal.pone.0188674 (PMC5695844; doi:10.1371/journal.pone.0188674)
Supplement: S2 File — (PDF) [file pone.0188674.s002.pdf]

PLOS ONE

RESEARCH ARTICLE

Multiscale adaptive analysis of circadian rhythms and intradaily variability: Application to actigraphy time series in acute insomnia subjects

Ruben Fossion[1,2]*, Ana Leonor Rivera[1,2], Juan C. Toledo-Roy[1,2], Jason Ellis[3], Maia Angelova[4]

1 Instituto de Ciencias Nucleares, Universidad Nacional Autónoma de México, 04510 Mexico City, Mexico, **2** Centro de Ciencias de la Complejidad, Universidad Nacional Autónoma de México, 04510 Mexico City, Mexico, **3** Faculty of Health and Life Sciences, Northumbria University, Newcastle Upon Tyne NE1 8ST, United Kingdom, **4** School of Information Technology, Melbourne Burwood Campus, Deakin University, Burwood VIC 3125, Australia

* ruben.fossion@nucleares.unam.mx

Abstract

Circadian rhythms become less dominant and less regular with chronic-degenerative disease, such that to accurately assess these pathological conditions it is important to quantify not only periodic characteristics but also more irregular aspects of the corresponding time series. Novel data-adaptive techniques, such as singular spectrum analysis (SSA), allow for the decomposition of experimental time series, in a model-free way, into a trend, quasiperiodic components and noise fluctuations. We compared SSA with the traditional techniques of cosinor analysis and intradaily variability using 1-week continuous actigraphy data in young adults with acute insomnia and healthy age-matched controls. The findings suggest a small but significant delay in circadian components in the subjects with acute insomnia, i.e. a larger acrophase, and alterations in the day-to-day variability of acrophase and amplitude. The power of the ultradian components follows a fractal $1/f$ power law for controls, whereas for those with acute insomnia this power law breaks down because of an increased variability at the 90min time scale, reminiscent of Kleitman's basic rest-activity (BRAC) cycles. This suggests that for healthy sleepers attention and activity can be sustained at whatever time scale required by circumstances, whereas for those with acute insomnia this capacity may be impaired and these individuals need to rest or switch activities in order to stay focused. Traditional methods of circadian rhythm analysis are unable to detect the more subtle effects of day-to-day variability and ultradian rhythm fragmentation at the specific 90min time scale.

OPEN ACCESS

Citation: Fossion R, Rivera AL, Toledo-Roy JC, Ellis J, Angelova M (2017) Multiscale adaptive analysis of circadian rhythms and intradaily variability: Application to actigraphy time series in acute insomnia subjects. PLoS ONE 12(7): e0181762. https://doi.org/10.1371/journal.pone.0181762

Editor: Shin Yamazaki, University of Texas Southwestern Medical Center, UNITED STATES

Received: December 16, 2016

Accepted: July 6, 2017

Published: July 28, 2017

Copyright: © 2017 Fossion et al. This is an open access article distributed under the terms of the Creative Commons Attribution License, which permits unrestricted use, distribution, and reproduction in any medium, provided the original author and source are credited.

Data Availability Statement: The present study is a secondary analysis of previously published data (DOI: 10.1098/rsif.2013.1112) that is available online http://rsif.royalsocietypublishing.org/content/11/93/20131112.figures-only.

Funding: We acknowledge financial support from DGAPA-PAPIIT of the Universidad Nacional Autónoma de México (project numbers IN106215, IV100116 and IA105017), the Consejo Nacional de Ciencia y Tecnología (project number CB-2011-01-167441) and the Newton Advanced Fellowship

awarded to R.F. by the Academy of Medical Sciences, through the UK Government's Newton Fund program. The funders had no role in study design, data collection and analysis, decision to publish, or preparation of the manuscript.

Competing interests: The authors have declared that no competing interests exist.

Introduction

Circadian rhythms are physical, mental and behavioral variations that follow an approximately 24-hour cycle. In the last few decades, it has become well established that most—if not all—physiological systems obey regular circadian rhythms and these are largely controlled by a central clock and several peripheral oscillators [1]. More recently, it has been observed that conditions, such as "healthy" and pathological ageing, illness and medication use, can influence the regularity and amplitude of the circadian rhythm. Consequently, the focus in the field of chronobiology shifted from a description of periodic cycles to the quantification of irregularities and the study of the mechanisms underpinning their disruption and normalization [2, 3].

One factor that has been shown to influence the sleep/wake circadian rhythm, albeit predominantly from data derived from animal studies [4–7], is poor psychological adjustment (i.e. stress, anxiety and depression). When extrapolating these findings to humans, cross-sectional observations have identified relationships between stress, anxiety and depression, and circadian disruption [8, 9]. Surprisingly however, few studies have extended this research to consider the role of stress-related sleep disruption (e.g., acute insomnia) in the relationship between poor psychological adjustment and circadian dysregulation, despite an intimate relationship being observed between stress and the onset of insomnia [10–14]. One reason for this may well be that although polysomnography (PSG) is considered the "gold standard" objective measurement strategy in sleep research, the ability of PSG to detect circadian abnormalities and, for that matter, insomnia is limited, due largely to practical reasons: its cost, coupled with the number of nights of recording that would be required to differentiate "normal" night-to-night variability in sleep/wake schedules from circadian disruption and insomnia. Similarly, whilst other methods more specific to measuring circadian rhythms, such as core body temperature and dim light melatonin onset (DLMO), offer a great degree of sensitivity, at a reduced cost, they tend to lack *ecological validity*, which means that they can be used in the lab but are more difficult to measure in ambulatory settings [15]. Moreover, the protocols used to collect this data, which tend to be invasive, are likely to confound the measurements traditionally used to quantify insomnia (e.g., difficulties initiating or maintaining sleep).

Actigraphy, the monitoring of physical activity patterns over many days up to several weeks, has been used as a cheaper, non-invasive and ecologically valid method to study sleep-wake patterns in humans. As such actigraphy may well be a more suitable method to examine circadian abnormalities in individuals with insomnia. Where the American Academy of Sleep Medicine (AASM) suggests actigraphy is not required for a diagnosis of insomnia, they do consider it an optional method to examine suspected circadian disruptions in this population [16, 17]. However, studies using actigraphy to determine whether circadian disruptions are a general feature of insomnia have been largely inconsistent [18, 19]. There may be two reasons for this lack of consistency: (i) either circadian disruption is not routinely a feature of insomnia by the time it has become chronic, or (ii) the methodology, or more likely, the statistical method of analysis, is not sensitive enough to identify small, albeit meaningful, levels of circadian disruption should they exist. Certainly, one of the reasons that actigraphy is presently not solely indicated for the diagnosis of any sleep disorder is due to problems identifying and eliminating artifacts in the data [20].

The traditional method to analyse circadian rhythms is cosinor analysis, which quantifies the 24-hour (24h), and other periodic cycles, by means of examining the degree of "fit" between the data and a user-defined model consisting of a superposition of cosine functions [21, 22]. However, experimental data where the statistical properties vary over time (non-stationary data), such as having a dominant trend [23–25], or time-varying amplitudes, frequencies or phases [26–28], are much harder or impossible to describe using models based on

these periodic functions. Another disadvantage of the cosinor method is that it is unable to detect rhythm fragmentation. With this in mind, the traditional approach, particularly for the analysis of actigraphy data, has been the measure of intradaily variability (IV), which is not model-based and hence is a nonparametric method. IV quantifies the frequency and the importance of transitions between periods of rest and activity, and for historical reasons is generally applied to hourly clustered data [2, 29, 30]. Although qualitatively different, the cosinor method and the IV method can be seen as complementary, where the focus of the former is the characterization of the 24h periodic aspects of the data, whereas the latter assesses the degree of rhythm fragmentation. Recently, more specialized techniques have been developed to study circadian rhythms (see Ref. [31] for a review). In particular, wavelets have been used to study circadian rhythms of nonstationary data [27, 28]. Wavelets however are, as with cosinor analysis, model based in the sense that the results obtained may depend on the particular wavelet basis function selected by the user. While continuous wavelet transforms may need an explicit prior detrending, discrete wavelet transforms are more effective in extracting time series components.

In the broader context of time-series analysis applied to physiology, it has been found that most physiological variables exhibit, what is often thought to be, spontaneous fluctuations. However, these fluctuations are often not random but according to Fourier spectral analysis or the more advanced detrended fluctuation analysis (DFA), behave as a fractal $1/f$ noise that might reflect the harmonious contribution of a wide variety of biological processes at multiple scales. Further, a breakdown of this power law often indicates an impoverishment where only a few single dominant processes contribute. One of the best studied examples of physiological time series is heart rate variability, where $1/f$ noise is interpreted as a reflection of youth and health, and a departure from this power law is often a signature of increased health risk due to ageing [32] or chronic-degenerative disease [33]. $1/f$ behaviour and its breakdown has been studied in actigraphy data in previous studies applied to ageing and dementia [34–36].

A long-standing problem in time-series analysis is the presence in the data of the nonstationarities mentioned above. The most recent developments in time-series analysis which take account of these nonstationarities are data-adaptive techniques such as singular spectrum analysis (SSA) [37–41], empirical mode decomposition (EMD) [42, 43] and nonlinear mode decomposition (NMD) [44, 45], which—with one rare exception [46]—have never been applied in the analysis of circadian rhythms, but may be particularly useful for examining sleep/wake circadian abnormalities in individuals with insomnia using actigraphy data. The basic idea of these data-adaptive techniques is to decompose a time series as a sum of modes that describe separately non-oscillating trend, (quasi-)periodic components and high-frequency noise. These techniques are nonparametric because, in contrast to the classical Fourier decomposition, the modes are not model dependent and do not need to be periodic sine or cosine functions. Instead the modes are derived from the data itself, they are not limited to a single time scale or a limited range of scales, but describe the data at all scales present. The lack of accessible specialized software to carry out data-adaptive analysis is the likely reason that these techniques, to date, have not been applied to circadian-rhythm research; fortunately, several open-source implementations have recently become available in multiple platforms such as Mathematica, MatLab, R, Python, etc., for SSA [47–50], EMD [51–54] and NMD [55]. Of the data-adaptive methods mentioned, in the present work, we prefer SSA because of its closeness to standard Fourier spectral analysis and the availability of graphical tools such as the scree diagram that can be interpreted as a generalization of the well-known Fourier power spectrum.

The aim of the present study was to undertake, for the first time, a systematic analysis of circadian rhythms, using data-adaptive time-series analysis, in individuals with acute insomnia.

SSA was applied to 1-week continuous actigraphy time series data in a sample of young adults with acute insomnia compared to age-matched healthy controls. SSA was chosen on the basis that it combines the different aspects of circadian analysis, ultradian rhythm fragmentation and scaling analysis in one single consistent framework, and not only reproduces but also improves the statistical results of the more traditional approaches. It was hypothesized that there would be small but meaningful differences in the sleep/wake circadian and/or ultradian rhythms between acute insomnia subjects and asymptomatic controls.

The paper is organized as follows. In the section Materials and Methods the details of the data set are given and the data analysis methods of cosinor, intradaily variability (IV) and singular spectrum analysis (SSA) are explained. The Results section presents the analysis of the 1-week actigraphy series with cosinor, IV and SSA. In the Discussion section, we interpret the results and we discuss the possible clinical implications. The results and interpretations are summarized in the Conclusion section. Finally, in the Appendix, some technical details of the SSA method are provided.

Materials and methods

Experimental actigraphy time series

We based our analysis on experimental actigraphy time series from the publicly available data of the publication of Ref. [56] of some of the authors of the present article. The data collection for the original analysis was approved by the University of Glasgow Ethics Committee. The previous article compared day- and night-time physical activity patterns of acute insomnia subjects with those of asymptomatic controls and included individuals of all age ranges. Actigraphy offers an objective measure of the level of physical activity using movement counts per sample time interval. In modern devices the movement counts are usually taken per 30-second or 1-minute basis, called "epochs", but typically a whole range of sample intervals is possible, from seconds to hours. In principle, resting and waking intervals can be distinguished as absence or low levels of activity vs. high levels of movement, respectively. The data was recorded with an Actiwatch device, worn at all times throughout day and night, for a period of 2 weeks. However, not all subjects completed the entire 2-week period; therefore, in the present study, we decided to study 1-week continuous day-and-night actigraphy time series. Each time series consists of activity counts, summed at $P = 1$min epochs. We focused on young adults (18–40yo), including 23 asymptomatic controls (28yo ± 6, 7 males and 16 females) and 18 acute insomnia subjects (25yo ± 6, 5 males and 13 females).

Cosinor

The traditional method to study the periodic behaviour of circadian rhythms is cosinor analysis [21, 22]. The cosinor approach is based on regression techniques and is also applicable to equidistant or non-equidistant time series $x(n)$ of N discrete data points,

$$x(n) = \{x_1, x_2, \ldots x_N\}. \qquad (1)$$

Given a specific value for period T, the procedure consists of fitting a continuous cosine function $y(t)$ to time series $x(n)$,

$$y(t) = M + A\cos(2\pi t/T + \phi). \qquad (2)$$

When exposed to the normal day-and-night cycle, this period can be expected to be $T \approx 24$h. Minimizing the summed square residual errors $e_n^2 = (x_n - y_n)^2$ for all data points $n = 1, 2, \ldots, N$, allows to find values for the parameters of the circadian cycle: the rhythm-adjusted mean or

mesor M, the amplitude A and the phase ϕ. Here, ϕ indicates the height of the cosine wave at the start of the monitoring; because each monitoring session can start at an arbitrary time of the day, the phase ϕ does not give any physiological information on the monitored individual. Instead, a more interesting variable is the *acrophase* ϕ_0 which can be defined as the time of day where the circadian cycle obtains its maximum, with respect to a fixed moment in time which is the same for all subjects, e.g. taking midnight as a reference, and which can be expressed as hours and minutes (hh:mm), or alternatively, as an angle (taking into account the relation $360^\circ = 24$hrs), relative to this reference time.

An important result in cosinor analysis is the coefficient of determination R^2, which compares the variance of the residual errors e_n around the fitted model y to the variance of the time series $x(n)$ around its average value $\langle x \rangle$,

$$R^2 = 1 - \frac{\mathrm{Var(e)}}{\mathrm{Var(x)}} \quad (3)$$

$$= 1 - \frac{\sum_{n=1}^{N} (x_n - y_n)^2}{\sum_{n=1}^{N} (x_n - \langle x \rangle)^2}, \quad (4)$$

such that R^2 is a measure for the fraction of the variance of the time series that can be explained by the model $y(t)$.

Intradaily variability

The cosinor approach is only applicable within very restrictive conditions where the data behaves approximately as a cosine function, and any non-sinusoidality of the time series limits the applicability of the method. There has been a lot of interest for alternative measures and methodologies to quantify the characteristics of circadian rhythms independently from user-defined functions and that therefore are called *nonparametric*. One popular nonparametric measure, applied in particular to actigraphy time series, is intradaily variability (IV), proposed in 1990 by Witting et al. [2] and reviewed recently in Ref. [29]. Because of limitations in available technologies when IV was originally proposed, the measure is traditionally applied to a time series $X(n)$ sampled at $P = 60$min intervals. It can be defined as,

$$\begin{aligned} \mathrm{IV} &= \frac{\mathrm{Var}(X')}{\mathrm{Var}(X)} \\ &= \frac{\sum_{n=2}^{N} (X_n - X_{n-1})^2/(N-1)}{\sum_{n=1}^{N} (X_n - \langle X \rangle)^2/N}, \end{aligned} \quad (5)$$

which corresponds to the variance of the derivative of the time series (differences of successive time-series values $X'(n) = X_n - X_{n-1}$ which fluctuate around zero, see Figs 5 and 6 in S1 File) with respect to the variance of the time series $X(n)$ around its mean $\langle X \rangle$. IV is a measure of the frequency and the importance of transitions between resting and activity periods, and how much these transitions contribute to the total variance of the time series.

Since the measure IV was introduced, battery and memory capacity of actigraphy equipment has improved in an important way such that data can be recorded at a wide variety of sampling intervals P instead of only at epochs of $P = 1$h, such that the $P = 60$min interval convention in itself has actually become a parameter. Any time series $x(n)$, such as the one of Eq (1), sampled originally at a high temporal resolution, e.g. $P = 1$min, can be resampled to new times series $X_P(n)$ of lower temporal resolution using different sample intervals $P > 1$min. In Ref. [30], it was found that considering intradaily variability IV(P) as function of the

resampling interval P, i.e., $\mathrm{IV}(P) = \mathrm{Var}(X'_P)/\mathrm{Var}(X_P)$, facilitated the comparison between different study populations; moreover, it was found that statistical differences between the different populations can maximize for sample intervals P that do not necessarily correspond to the arbitrary convention of $P = 60$min. Here, however, we argue that $\mathrm{IV}(P)$ is a composite function, where nominator $\mathrm{Var}(X'_P)$ and denominator $\mathrm{Var}(X_P)$ can depend on P each in their own way. Here, we redefine the function $\mathrm{IV}(P)$ as follows,

$$\mathrm{IV}(P) = \frac{\mathrm{Var}(X'_P)}{\mathrm{Var}(x)} \tag{6}$$

using the variance of the original time series $x(n)$, which is a constant, as the denominator, such that $\mathrm{IV}(P)$ is a simple and unequivocal function of resampling interval P (Fig 7 in S1 File compares different normalization conventions).

Singular spectrum analysis (SSA)

SSA has been discussed in detail in a number of textbooks [37–39], a short and very accessible introduction can be found in Ref. [40], whereas a larger and very complete review article is Ref. [41]. In brief, SSA can be explained as a 3-step process: (i) the time series is transformed into a matrix which represents the underlying phase space of the time series, (ii) singular value decomposition (SVD) is applied to decompose this matrix as a sum of elementary matrices, or—equivalently—to decompose the original phase space in a superposition of "sub phase-spaces", and (iii) each of the elementary matrices or "sub phase-spaces" is transformed back into a time-series component. Unlike Fourier analysis which expresses a time series as a sum of predefined sine and cosine functions, SSA can be considered to be *data-adaptive* or *model-independent* because the basis functions are generated from the data itself. It can be shown that the sum of all time-series components is identical to the original time series. Below, a summary is given of the most important outcomes of SSA analysis, whereas some technical details are briefly discussed in the Appendix.

When applying SSA to a discrete time series $x(n)$ with length $n = 1, \ldots, N$, see Eq (1), a particular window length L must be chosen as an initial parameter, with $2 \leq L \leq N/2$, which allows to fix the number of components r into which the time series will be decomposed,

$$x(n) = \sum_{k=1}^{r} \sigma_k g_k(n), \tag{7}$$

where $g_k(n)$ are time-series components, σ_k are *singular values* that serve as weights for the components and $r \leq \min(K, L)$ with $K = N - L + 1$. Only (quasi-)periodicities with average length $T \lesssim L$ will be resolved into separate time-series components, whereas those with lengths $T > L$ will be absorbed in the trend component. One can chose L as a multiple of the (average) periodicity of the data, i.e. $L = mT$, where m is an integer number. In the case of circadian data the obvious choice would be $L = T = 24$h $= 1140$min. It can be shown that in the limit for $L \rightarrow N/2$, SSA converges to Fourier spectral analysis [41], where a time series is always decomposed as the superposition of $N/2$ independent oscillators (Nyquist theorem). Whereas the Fourier power spectrum of a quasiperiodic time series with average period T would correspond to a broad gaussian peak around some central frequency $f = 1/T$, intuitively, it can be understood that for an adequate choice of the parameter L the neighbouring Fourier components of this broad gaussian peak can be "compressed" within a single SSA component.

One of the main results of SSA analysis is the so-called *scree diagram* that visually represents the *partial variances* $\lambda_k = \sigma_k^2$, ordered according to magnitude from the most to the least dominant, where λ_k can be interpreted as the variance of the "sub phase-space" of time-series

component $g_k(n)$ and where $\lambda_{tot} = \sum_{k=1}^{r} \lambda_k$ is the total variance of the phase space of the original time series $x(n)$. The dominant partial variance λ_1, associated with component $g_1(n)$, usually corresponds to the trend. Dominant periodicities can be recognized as "steps" in the scree diagram, i.e., two successive partial variances λ_k and λ_{k+1} that are nearly degenerate and clearly distinguishable from the neighbouring partial variances, and where the corresponding components $g_k(n)$ and $g_{k+1}(n)$ are the Fourier equivalents of a sine and cosine function with the same frequency. Higher-order partial variances λ_k tend to have values that decrease gradually and continuously with k, indicating that at these scales it is impossible to distinguish any individual time-series components $g_k(n)$ that can be assigned physical significance.

Results

Visual inspection of time series

In Fig 1, one week of continuous actigraphy measurement is compared for a specific control subject and a subject with acute insomnia. In both cases, the night-time resting period can be roughly recognized as a 6–8h interval of minimal physical activity. Comparing the visual aspects of the time series shown, it can be observed that the control subject goes to bed and gets up earlier than the acute insomnia subject, but the total duration of the resting period appears to be similar between both subjects. A more subtle difference is that the time series of the acute insomnia subject seems to be more "spiky" or intermittent, whereas the time series of the control subject is characterized by more and longer intervals with continuous activity at a more or less constant intensity level. In Fig 2, the average 24h actigraphy profile is compared for the population of asymptomatic controls and the population of acute insomnia subjects. Here, and in what follows, statistical significance was calculated using a Kruskal-Wallis non-parametric test (IBM SPSS software version 22), with an a priori significance level of $p = 0.05$. The average 24h profile shows significant differences between the two populations for the rest-to-wake (07:22–09:01) and wake-to-rest transitions (23:01–24:00).

Cosinor

Values for the period T were calculated for each subject individually and were determined in order to maximize the amplitude A of the cosinor fit of Eq (2) (see Fig 2 in S1 File). For all subjects, period $T \approx 24$h, with a somewhat larger dispersion around the ideal period $T_0 = 24\text{h} = 1440\text{min}$ for the subjects with acute insomnia than for the controls, but without statistical significance, see Table 1 and Fig 3. Subsequently, values for the other circadian parameters were obtained by regression analysis: mesor M, amplitude A and phase ϕ, see Table 1 and Fig 4. Also values for the coefficient of determination R^2 and for the acrophase ϕ_0 were calculated, see Table 1 and Fig 3. Mesor M, period T and amplitude A are constants. On the other hand, when $\Delta T = T - T_0 \neq 0$, these differences ΔT will accumulate day after day leading to a linear delay ($\Delta T > 0$) or advance ($\Delta T < 0$) of acrophase ϕ_0 as a function of time, see Fig 5. Therefore, in Table 1 and Fig 3, 7-day average values are given for ϕ_0. R^2 values are slightly larger for the control group than for the acute insomnia group, but without statistical significance. All circadian parameters are similar for both populations with exception of the mean acrophase ϕ_0, see Table 1 and Fig 3, and indicates that the time of day with maximum activity is significantly delayed with about 1.5hrs in acute insomnia subjects with respect to the controls.

Intradaily variability (IV)

In Fig 6, results are shown for the nonparametric measure of intradaily variability IV(P) as a function of the sampling interval P, according to Eq (6), comparing the population of

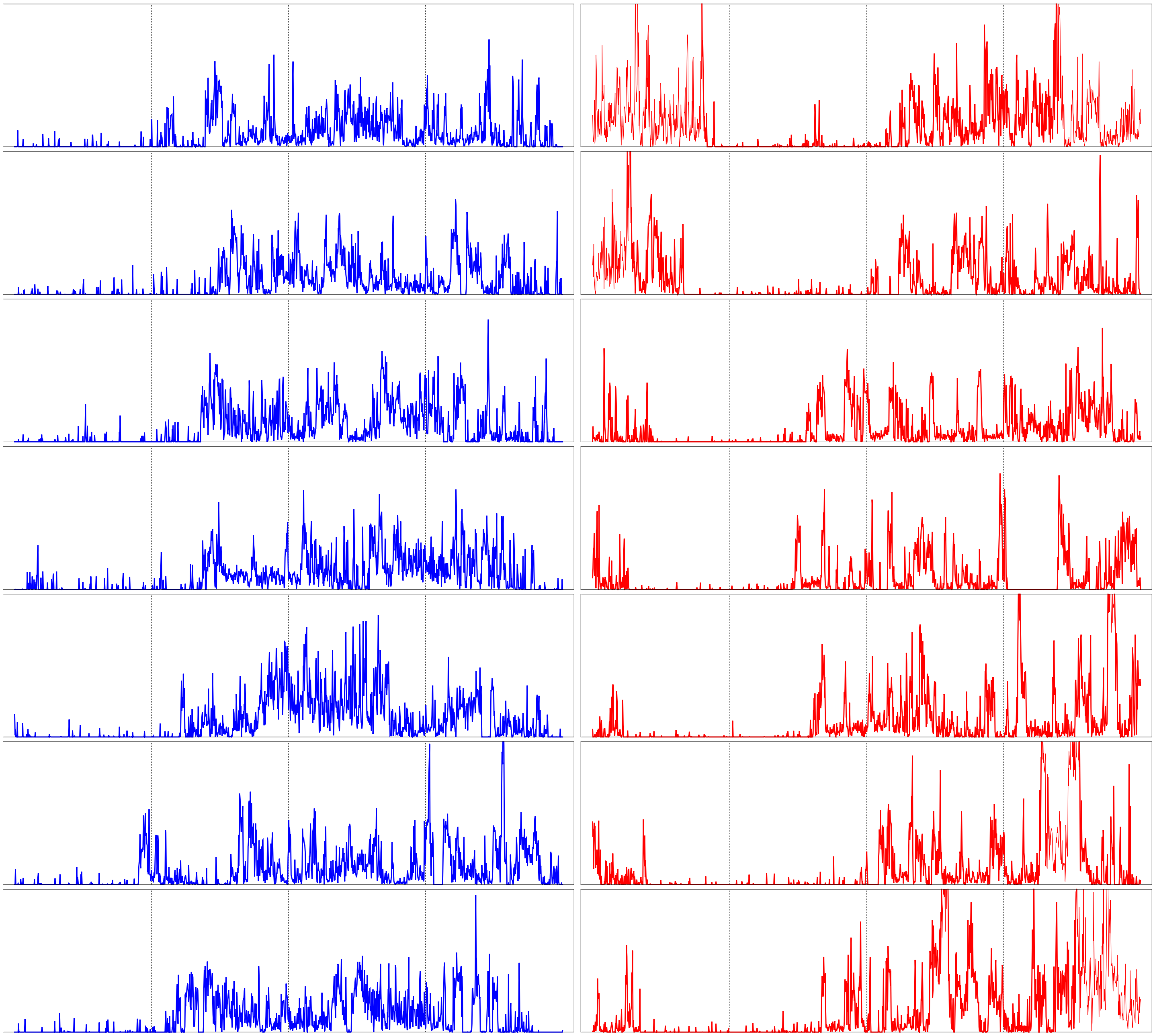

Fig 1. 1-week continuous actigraphy time series. Shown for a specific control subject (female, 24yo, left-hand panels) and a subject with acute insomnia (male, 22yo, right-hand panels). Shown for 7 successive days (24h per panel), from midnight till midnight, with vertical gridlines at 6h intervals at 00:00, 06:00, 12:00, 18:00 and 24:00 hours. Vertical scale is identical for both subjects, from 0 to 3000 movement counts per minute.

https://doi.org/10.1371/journal.pone.0181762.g001

asymptomatic controls and the acute insomnia subjects. For both populations, there is a local maximum near $P = 500$min; for the population with acute insomnia, there is another local maximum near $P = 20$min. It should be noted that the time scales of these two relative maxima fall outside of the scale $P = 60$min as considered traditionally for IV analysis. The variability of the maximum at $P = 500$min is due to the circadian cycle of 24h which is the only oscillation slow enough to be observed in units of $P = 500\text{min} \approx 8$hrs. The variability of the maximum at

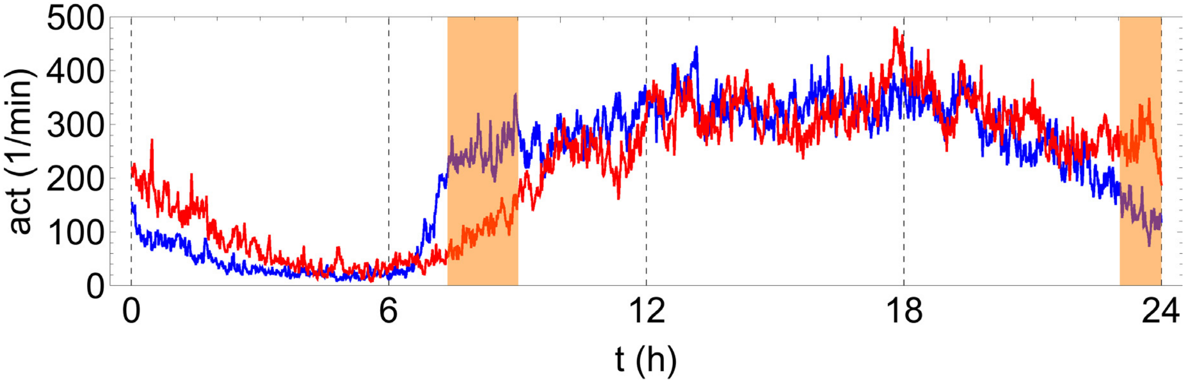

Fig 2. Mean 24h profile of actigraphy time series. Averaged over 7 days and over all control subjects (blue curve) and over all subjects with acute insomnia (red curve). Vertical gridlines at 6h intervals. Significant ($p < 0.05$) minute-to-minute differences are observed between the 2 groups for rest-to-wake (07:22–09:01) and wake-to-rest (23:01–24:00) transitions (orange shading).

https://doi.org/10.1371/journal.pone.0181762.g002

$P = 20$min for the population with acute insomnia is related to an ultradian variation with a period that is a multiple of this particular sample interval. According to the Kruskal-Wallis nonparametric test there are no significant statistical differences between both populations for the measure $\mathrm{IV}(P)$.

Singular spectrum analysis (SSA)

All calculations have been carried out with parameter value $L = 1440$min, the results however are largely independent of the specific value of L. An important result in SSA analysis is the scree diagram of ordered fractional partial variances $\lambda_k/\lambda_{\mathrm{tot}}$ which graphically represents the relative importance of each of the time-series components in the original time series. A scree diagram is usually represented in log-log scale, see Fig 7, and in the present case one can distinguish a dominant $\lambda_1/\lambda_{\mathrm{tot}}$ corresponding to a non-oscillating trend or mesor component $g_1(n)$, then—one order of magnitude below—come $\lambda_2/\lambda_{\mathrm{tot}}$ and $\lambda_3/\lambda_{\mathrm{tot}}$ with very similar values corresponding to the periodic time-series components $g_2(n)$ and $g_3(n)$ that together constitute the circadian rhythm, and finally—again one order of magnitude below—a long tail $k \geq 4$ of smaller fractional partial variances $\lambda_k/\lambda_{\mathrm{tot}}$ with gradually diminishing values that correspond to time-series components $g_k(n)$ at ultradian time scales (see Figs 8 and 9 in S1 File for a graphical representation of some of these time-series components where the average period has

Table 1. Parameters of the circadian rhythm according to cosinor analysis.

	Controls	Acute insomnia	p
T	1437 ± 20	1435 ± 22	0.733
M	216 ± 56	218 ± 63	0.895
A	178 ± 60	178 ± 61	0.979
mean(ϕ_0)	232 ± 22	253 ± 27	0.017 (*)
	15 : 28 ± 01 : 28	16 : 52 ± 01 : 48	
R^2	0.14 ± 0.06	0.12 ± 0.05	0.446

Period T (min), mesor M (1/min), amplitude A (1/min), 7-day average acrophase ϕ_0 expressed in degrees (°) and in hours and minutes (hh:mm) after midnight, and coefficient of determination R^2. Values given in the table are the population average and the standard deviation. Statistical significance (*) is considered for $p < 0.05$ according to a nonparametric Kruskal-Wallis test.

https://doi.org/10.1371/journal.pone.0181762.t001

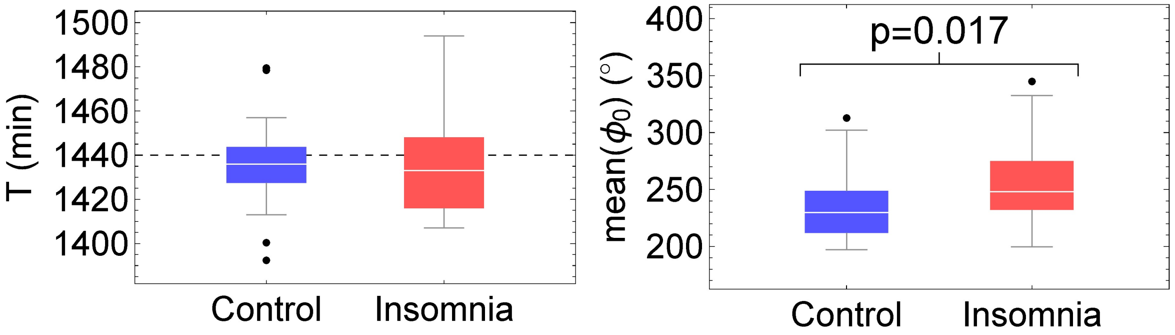

Fig 3. Circadian parameters according to cosinor analysis. Box-whisker plots for period T with respect to the ideal period of T_0 = 24h = 1440min (dashed horizontal line), and 7-day average acrophase ϕ_0 (°).

https://doi.org/10.1371/journal.pone.0181762.g003

been calculated to corroborate the identification of a component being ultradian, circadian or trend). Fractional partial variances of the mesor and the circadian components are similar for the controls and the subjects with acute insomnia. Interestingly, in the case of the control subjects, the fractional partial variances at the ultradian scales appear to follow a single power law $\lambda_k \propto 1/k^{\gamma}$ with $\gamma \approx 1$, for the whole range $0.78 \leq \log_{10}(k) \leq 3.0$. On the other hand, in the case of the subjects with acute insomnia, this power law appears to be broken because of an increased variability near $k = 30$ (or $\log_{10}(k) = 1.5$) corresponding to an average frequency $\langle f \rangle = 1/60–1/90$min, and results in a crossover behaviour between different power laws with γ_1 at larger scales before the crossover ($0.8 \leq \log_{10} k \leq 1.5$) and γ_2 at smaller scales after the

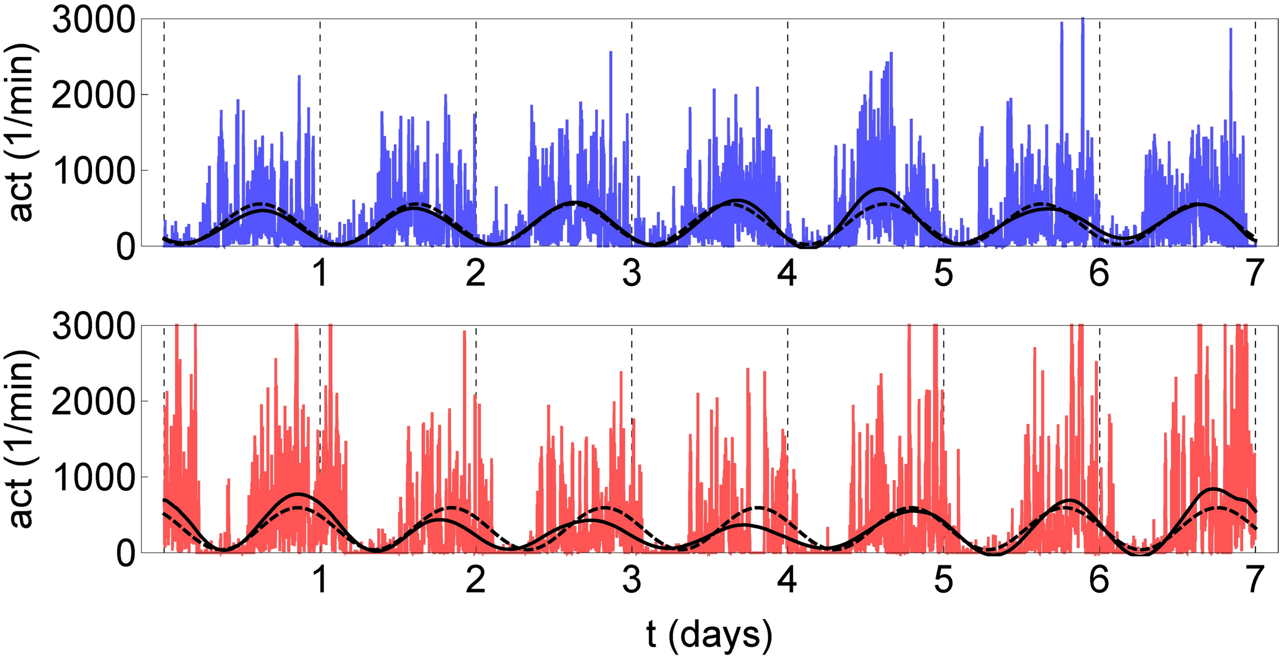

Fig 4. Circadian component of actigraphy data. Shown for the control (upper panel) and the acute insomnia subject (bottom panel) of Fig 1. The circadian rhythm has been fitted using the model-based cosinor method according to Eq (2) (dashed curve) and the data-adaptive SSA method using the periodic components $g_2(n)$ and $g_3(n)$ of Eq (7) (full curve). Vertical gridlines at midnight.

https://doi.org/10.1371/journal.pone.0181762.g004

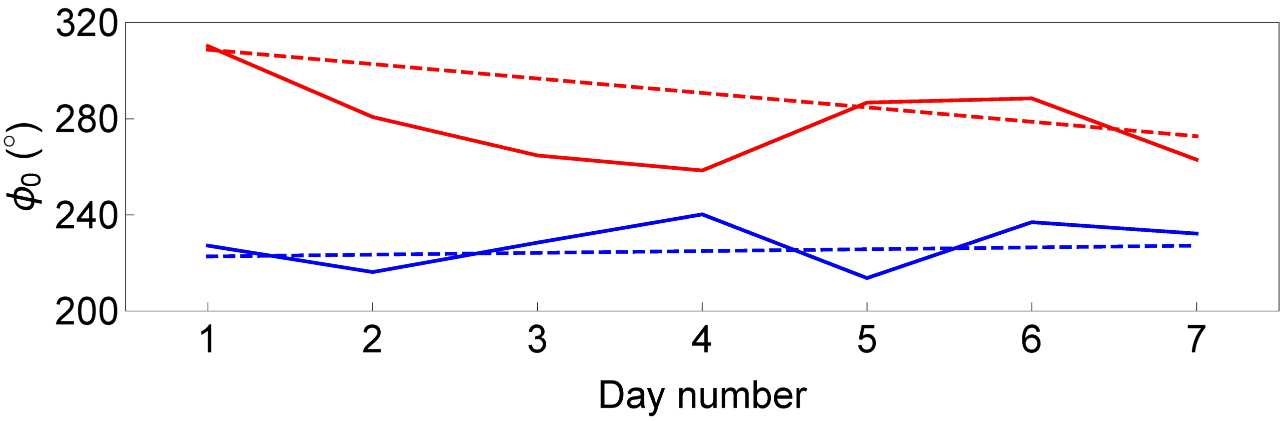

Fig 5. Acrophase $\phi_0(t)$ as a function of time. Results for the subjects of Fig 1. Within the cosinor approach (dashed lines), the deviation from the ideal 24h circadian cycle is small for the control subject, $\Delta T = +3$min, such that $\phi_0(t)$ is rather constant (blue dashed line), whereas for the subject with acute insomnia the circadian deviation is larger, $\Delta T = -24$min, and $\phi_0(t)$ is a downsloping linear function (red dashed line). SSA (continuous lines) allows to calculate the day-to-day variability of acrophase $\phi_0(t)$, and are found to fluctuate around the average behaviour obtained within the cosinor approach.

https://doi.org/10.1371/journal.pone.0181762.g005

crossover ($1.6 \leq \log_{10} k \leq 2.0$), see Table 2 and Fig 8. The differences in scaling behaviour between controls and insomniacs are statistically significant, and for insomniacs also the increased variability in the range $\langle f \rangle$ = 1/60–1/90min and the scaling parameters γ_1 and γ_2 before and after the crossover; these differences are valid at the level of the individual subjects as can be seen from Fig 8.

The circadian cycle is well described by the periodic components g_2 and g_3, see Fig 4, and according to the R^2 measure the description is equally good for the controls as for the subjects with acute insomnia, see Table 2. SSA describes the day-to-day variations in the circadian parameters of mesor M, amplitude A, period T and acrophase ϕ_0, see Fig 4. Therefore, in order to compare the values of these parameters for the two populations, 7-day average values are presented in Table 2. Additionally, statistical measures such as the standard deviation (SD), coefficient of variation (CV = SD/mean), skewness (Skew) and kurtosis or "peakedness" (Kurt) are listed, to give information on the day-to-day variations for all individuals of each

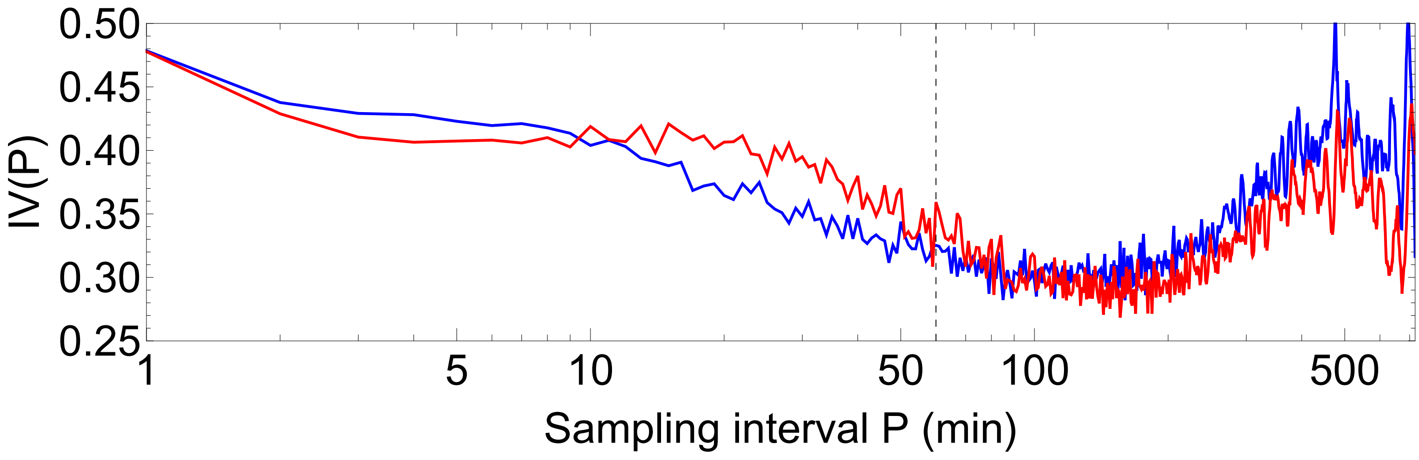

Fig 6. Intradaily variability $IV(P)$ as a function of sample interval. Calculated according to Eq (6). Shown for the population of asymptomatic controls (blue) and acute insomnia subjects (red). Vertical gridline at traditional sample interval $P = 60$min.

https://doi.org/10.1371/journal.pone.0181762.g006

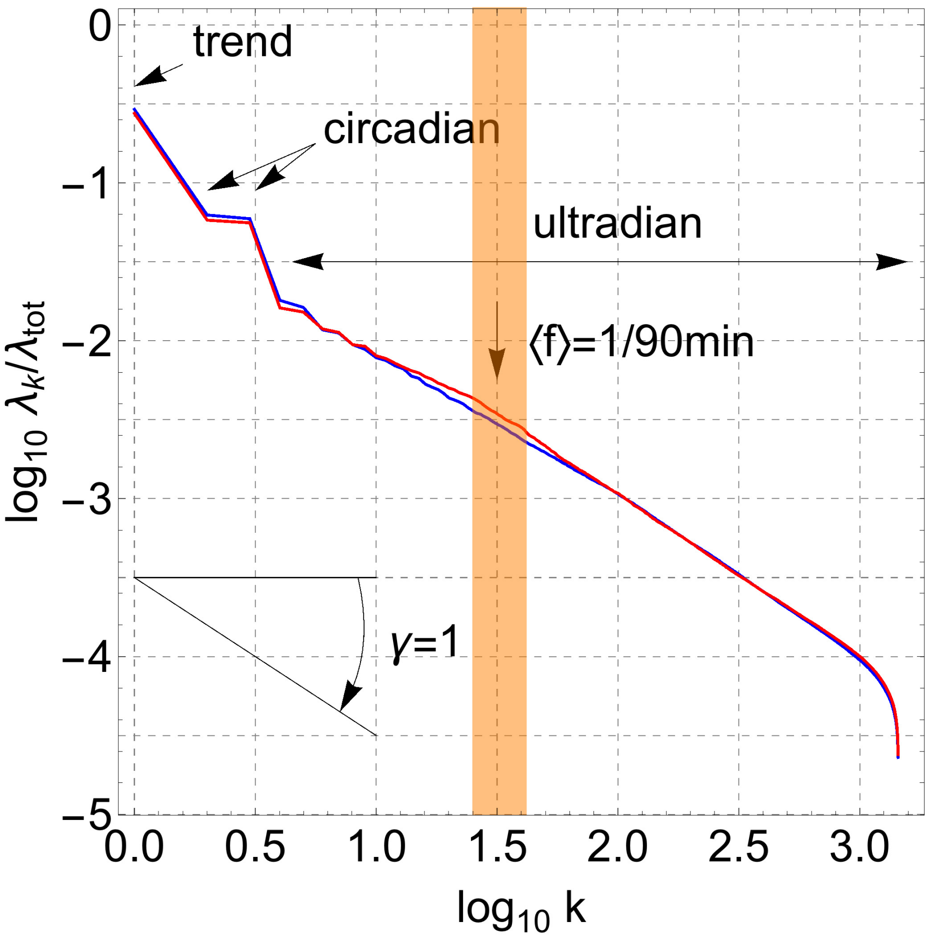

Fig 7. SSA scree diagram of ordered fractional partial variances. Fractional partial variances λ_k/λ_{tot} ordered from the most dominant λ_1/λ_{tot} corresponding to the non-oscillating trend or mesor, and $(\lambda_2 + \lambda_3)/\lambda_{tot}$ corresponding to the periodic circadian cycle, down to higher-order λ_k/λ_{tot} with $k \geq 4$ corresponding to ultradian fluctuations. In the case of the control subjects (blue), fractional partial variances follow a power law $\lambda_k/\lambda_{tot} \propto 1/k^{\gamma}$ with power-law exponent $\gamma \approx 1$ (negative of the slope of the scree diagram in log-log scale), whereas in the case of the acute insomnia subjects (red) this power law is broken because of an increased variability around $\langle f \rangle = 1/90$min, these differences are statistically significant in the range $1.4 \leq \log_{10} k \leq 1.6$ (shaded region).

https://doi.org/10.1371/journal.pone.0181762.g007

population. With respect to the 7-day average values of the circadian parameters, there are statistically significant differences only for the acrophase ϕ_0, where the subjects with acute insomnia show a 1.5hr delay in the moment of the day with maximum activity. With respect to the day-to-day variations, there are significant differences for acrophase ϕ_0 and amplitude A. The kurtosis of the ϕ_0 values over 7 successive days is Kurt$\approx$ 3 for the controls, indicative of a gaussian distribution, whereas Kurt$\approx$ 2 for the acute insomnia subjects, which indicates a platykurtic distribution, see Fig 9. Although the standard deviation of successive ϕ_0 values is similar for controls and acute insomnia subjects, in the former case there is a large spread in SD values between subjects, whereas because of the platykurtic distribution more homogeneous results are obtained for SD for the acute insomnia subjects. The standard deviation of amplitude A over successive days are significantly larger for the acute insomnia subjects than for the control subjects, and this difference is even more explicit for the coefficient of variation which expresses SD as a fraction of the average value, see Fig 9.

Table 2. Parameter values of the circadian and ultradian rhythms according SSA analysis.

	Controls	Acute insomnia	p
mean(T)	1432 ± 35	1429 ± 24	0.572
mean(M)	217 ± 57	218 ± 64	1.00
mean(A)	190 ± 55	186 ± 60	0.431
mean(ϕ_0)	234 ± 23	253 ± 25	0.013 (*)
	15 : 36 ± 1 : 32	16 : 52 ± 1 : 40	
SD(ϕ_0)	26 ± 16	23 ± 9	0.415
CV(ϕ_0)	0.11 ± 0.06	0.09 ± 0.03	0.202
Skew(ϕ_0)	0.28 ± 0.97	0.09 ± 0.54	0.378
Kurt(ϕ_0)	2.84 ± 0.87	2.21 ± 0.60	0.015 (*)
SD(A)	51 ± 17	69 ± 27	0.031 (*)
CV(A)	0.28 ± 0.12	0.37 ± 0.10	0.010 (*)
Skew(A)	0.12 ± 0.72	0.31 ± 0.61	0.535
Kurt(A)	2.27 ± 0.67	2.34 ± 0.72	0.588
γ_1	0.85 ± 0.17	0.73 ± 0.21	0.04 (*)
γ_2	0.86 ± 0.15	1.02 ± 0.22	0.04 (*)
R^2	0.20 ± 0.06	0.20 ± 0.05	0.979

Parameter values of the circadian cycle for period T (min), mesor M (1/min), amplitude A (1/min), acrophase ϕ_0 in degrees (°) and hours and minutes (hh:mm) after midnight, and coefficient of determination R^2. Values given in the table are the population average and the standard deviation. (*) Considered to be significant ($p < 0.05$) according to a nonparametric Kruskal-Wallis test.

https://doi.org/10.1371/journal.pone.0181762.t002

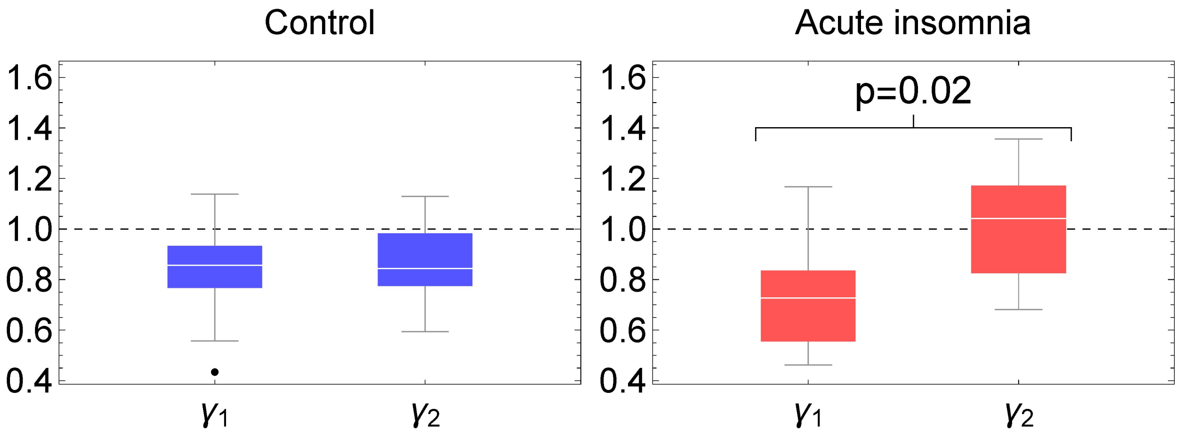

Fig 8. Fractal scaling parameters according to SSA analysis. Box-whisker plots of the scaling exponent of the power law $\lambda_k \propto 1/k^\gamma$ at larger scales (exponent γ_1) and at smaller scales (exponent γ_2) before and after $\log_{10} k = 1.5$, shown for the control subjects which follow a single power law (blue) and subjects with acute insomnia which exhibit a crossover (red).

https://doi.org/10.1371/journal.pone.0181762.g008

Discussion

The focus of circadian-rhythm research has shifted over the years from the demonstration of the existence of a regular circadian modulation of physiological variables to more recent investigations of how these rhythms degenerate with ageing and disease. The use of circadian analysis as a diagnostic tool is becoming increasingly important to quantify deviations from regularity in circadian cycles. It has been hypothesized that insomnia might be related to

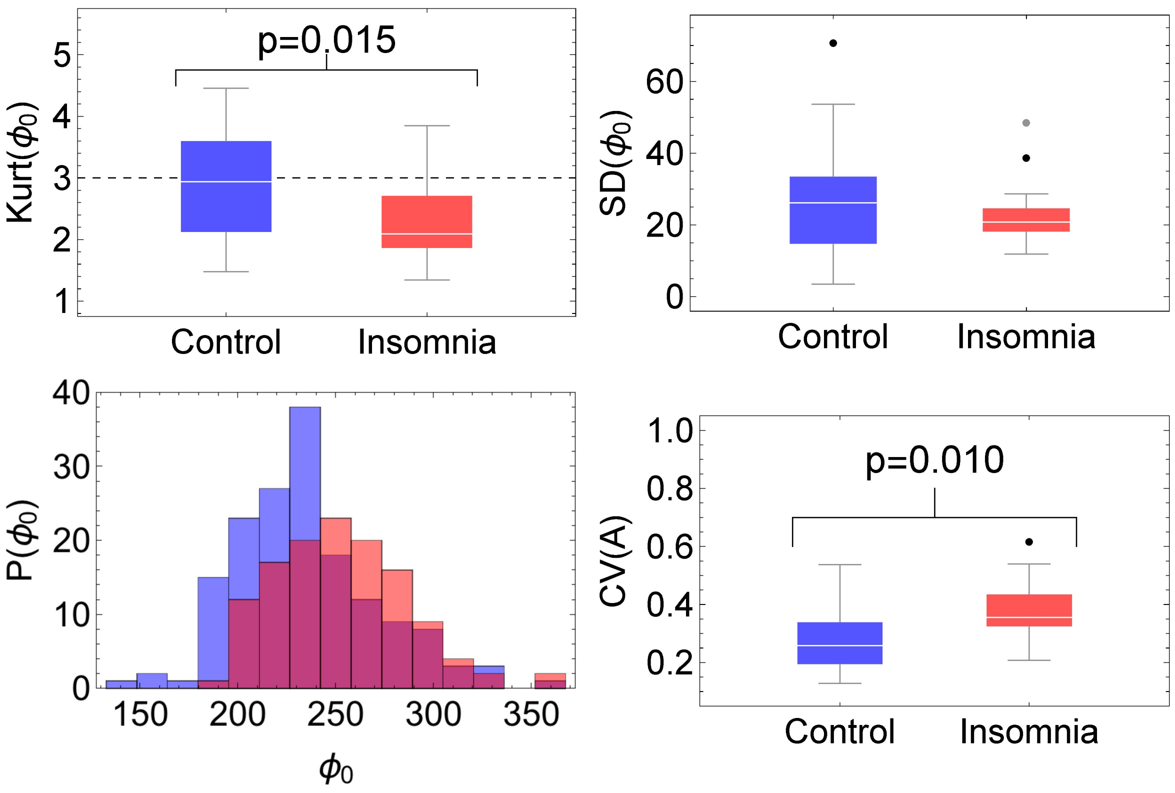

Fig 9. Day-to-day variability of circadian parameters according to SSA analysis. Histogram of acrophase ϕ_0, box-whisker plots of standard deviation (SD) and kurtosis (Kurt) of acrophase ϕ_0 (with Kurt = 3 for a gaussian distribution as a reference), and coefficient of variation CV = SD/mean of amplitude *A*, for the controls (blue) and the acute insomnia subjects (red).

https://doi.org/10.1371/journal.pone.0181762.g009

alterations, albeit small, in circadian and ultradian rhythms, but after many years of study this topic remains an open problem. In the present article, we studied 1-week continuous actigraphy time series in young adults with acute insomnia in comparison with age-matched control subjects, using more traditional methods such as cosinor analysis, intradialy variability (IV) and the novel data-adaptive time-series technique of singular spectrum analysis (SSA).

With respect to the average parameters of the circadian cycle, traditional cosinor analysis detects a small but significant delay of about 1.5hrs in the acrophase ϕ_0 in subjects with acute insomnia compared to those in the control subjects, whereas the other parameters of period *T*, mesor *M* and amplitude *A* are similar in both populations. These results were confirmed by SSA analysis, which is not surprising because the description of the circadian rhythm is very similar for cosinor and SSA, as illustrated in Fig 4 and quantified by the rather large Pearson and Spearman's rank correlation coefficients *r* and ρ, see Table 3. However, comparing the

Table 3. Correlation between cosinor and SSA circadian components.

	Controls	Acute insomnia
Pearson's *r*	0.83 ± 0.10	0.81 ± 0.09
Spearman's ρ	0.84 ± 0.10	0.84 ± 0.08

Pearson's correlation coefficient *r* and Spearman's rank correlation coefficient ρ for the description of the circadian cycle of the asymptomatic controls and the subjects with acute insomnia. Values given in the table are the population average and the standard deviation.

https://doi.org/10.1371/journal.pone.0181762.t003

coefficients of determination R^2 for both methods of analysis, see Tables 1 and 2, it can be seen that SSA improves the description of the circadian cycle in an important way, mainly due to the inclusion of day-to-day variability in the SSA description whereas cosinor only evaluates average properties of the circadian cycle.

Regarding the day-to-day variability of the circadian cycle, SSA detects a significantly larger variability for amplitude A for the subjects with acute insomnia than for the control subjects, whereas for acrophase ϕ_0 a gaussian distribution is found for the controls and a more platykurtic distribution for those with acute insomnia. A possible interpretation may be that acute insomnia does not affect the mean activity level over 1 week. This suggestion is further supported as both cosinor and SSA agree that mesor M and amplitude A on the average are similar for both populations, see Tables 1 and 2. On the other hand, in individuals with acute insomnia, the physical activity level for a specific day may depend on the previous nights' sleep quality, whereby a night of poor sleep is related to less active behaviour the following day, potentially as a volitional method to recuperate. In contrast, the control subjects who do not have sleeping problems appear to be able to maintain more constant activity levels over successive days. The difference in the distribution of acrophase ϕ_0 between both populations may be related to the deviations $\Delta T = T - T_0$ in the length of the circadian cycle from the ideal period $T_0 = 1440$min, see Fig 3. Although there are no statistical significant differences between both groups for the deviations from the ideal circadian cycle ΔT, in the acute insomnia group these somewhat larger deviations may accumulate and can result in a trend for ϕ_0 over successive days. Fig 5, which shows the acrophase as a function of time $\phi_0(t)$, suggests that the cosinor method describes the linear trend of the day-to-day variability of the acrophase as calculated with SSA. Trends tend to lower the kurtosis of time series, as can be verified easily by adding a linear trend to random gaussian noise (Kurt = 3), which reduces the kurtosis to more platykurtic values (Kurt<3), see Fig 14 in S1 File.

For the ultradian cycles, using the nonparametric method of intradaily variability IV(P) as a function of sample interval P, we found an increased variability in the range $10 < P < 50$min for the subjects with acute insomnia indicating enhanced ultradian rhythms with respect to the healthy controls and with periods that are a multiple of these particular sample intervals. Although not significant within the approach of IV, these enhanced ultradian rhythms probably reflect the more intermittent and "spiky" behaviour in the actigraphy time series of individuals with acute insomnia, as discussed before in relation with Fig 1. SSA is very similar to intradaily variability, it also breaks down the time series at different scales to quantify how much each scale contributes to the total variance of the time series, but with the additional feature that the time-series components can be extracted to study their dynamics in more detail. Using SSA analysis, we found a similar increase of variability for the subjects with acute insomnia with respect to the controls at a frequency range of $\langle f \rangle = 1/60\text{–}1/90$min, and in this case the differences were statistically significant. For the healthy controls, there is no indication of enhanced variability in ultradian cycles at any frequency range, instead the fractional partial variances $\lambda_k/\lambda_{\text{tot}}$ appear to behave as a $1/f$ power law over the whole range of ultradian scales. In subjects with acute insomnia this power law appears to be broken and a crossover can be seen between a less steep behaviour before and a steeper behaviour after the characteristic frequency of $\langle f \rangle = 1/60\text{–}1/90$min. We checked the SSA results with Fourier spectral analysis and DFA, see Figs 1, 13 and 15 in S1 File. Fourier reproduced all features of the SSA analysis but without statistically significant differences between the 2 populations. Because of technical limitations, DFA analysis can only be applied to day-time or night-time fragments and not to 1-week continuous time series, and DFA confirms the scaling results of SSA for smaller scales with significant differences between the controls and those with acute insomnia. The interpretation may be as follows. On the one hand, the particular frequency range $\langle f \rangle = 1/60\text{–}1/90$min

is reminiscent of Kleitman's basic rest-activity cycle (BRAC) model [57], which hypothesizes that the circadian 24h cycle can be subdivided into shorter ultradian oscillations that during sleep manifest as REM-nonREM cycles of ≈90min and during wakefulness in ≈90min fluctuations in cortical alertness and sleep propensity [58]. These 90min oscillations are not observed in the actigraphy data of asymptomatic subjects during their daily routine [59], but there is evidence that these rhythms become dominant in experimental conditions with sleep deprivation [60, 61], isolation [62], artificial 90min days [63] and when putting people on learning schedules of 90min [64–66]. On the other hand, fractal $1/f$ behaviour has been previously observed in actigraphy data using DFA in the context of ageing and dementia [34–36]. In physiology, heart rate variability is one of the best known examples of $1/f$ noise, and is interpreted as the harmonious contribution of many biological processes at multiple scales [67]; whereas a deviation from the $1/f$ power law is interpreted as an impoverishment and a predominance of a few single contributing processes [32, 33]. In essence, in healthy controls, the routine of a typical day may be composed of many different activities with multiple durations and intensities, whereas in subjects with acute insomnia the modulation of activity by BRAC 90min attention cycles or sleep-propensity cycles can make it more difficult to perform longer-term continuous activities.

Conclusion

The aim of the present study was to examine circadian rhythms in individuals with acute insomnia using a database of 1-week continuous actigraphy data of young adults with acute insomnia and age-matched asymptomatic controls. It was hypothesized that insomnia is associated with alterations in circadian and ultradian cycles. For the first time a systematic analysis was undertaken which employed a data-adaptive time-series technique, singular spectrum analysis (SSA). The findings suggest that this new approach is able to reproduce, improve and combine within a single consistent framework the results of the complementary traditional approaches of cosinor analysis and nonparametric intradaily variability (IV). Whereas the majority of the circadian parameters such as mesor, period and amplitude were similar for both populations, a small but significant delay in the acrophase for subjects with acute insomnia was observed. Moreover, alterations in the day-to-day variability of acrophase and amplitude in the acute insomnia population were also noted. At ultradian scales, for healthy controls, actigraphy data appears to behave as fractal $1/f$ noise, which indicates that the routine of a typical day does consist of many activities with multiple durations and intensities. In the case of acute insomnia subjects, this power law breaks down because of a significantly higher intradaily variability around the average frequency of 1/60–1/90min. This finding is reminiscent of Kleitman's basic rest-activity cycle (BRAC) model, which may indicate that 90min cycles in attention levels and/or sleep-propensity cycles modulate the activity level in subjects with acute insomnia interfering with the capacity to carry out long-term continuous activities. The more detailed aspects of day-to-day variability and ultradian statistics only became apparent using our new method based on SSA, whereas the traditional methods were unable to detect these effects. This more sensitive analysis in the present study further contributes to our understanding of the role of circadian disruption in acute insomnia.

Appendix: Technical details on the SSA method

Let $x(n)$ of Eq (1) be a discrete time series. SSA uses a parameter L as a window length, or *embedding dimension*, to embed the time series in a phase space which is represented as a so-called *trajectory matrix* $\mathbf{X}$. A sliding window $W_i = (x_i, x_{i+1}, \ldots x_{i+L-1})$ is passed over the time

series using a unit step size, $\Delta i = 1$, such that,

$$\mathbf{X} = \begin{pmatrix} W_1 \\ W_2 \\ W_3 \\ \vdots \\ W_K \end{pmatrix} = \begin{pmatrix} x_1 & x_2 & x_3 & \dots & x_L \\ x_2 & x_3 & x_4 & \dots & x_{L+1} \\ x_3 & x_4 & x_5 & \dots & x_{L+2} \\ \vdots & \vdots & \vdots & \ddots & \vdots \\ x_K & x_{K+1} & x_{K+2} & \dots & x_N \end{pmatrix}, \tag{8}$$

with $K = N - L + 1$. By construction, the trajectory matrix **X** is of Hankel type, i.e. each ascending diagonal has equal elements. Applying SVD to matrix **X** allows to identify new data-generated basis states $\vec{u}_k$ and $\vec{v}_k$ to re-express the data more meaningful physically, and in particular to decompose matrix **X** in a unique and exact way as a sum of elementary matrices, see Ref. [68],

$$\mathbf{X} = \mathbf{U}\Sigma\mathbf{V}^T = \sum_{k=1}^{r} \sigma_k (\vec{u}_k \vec{v}_k^T) = \sum_{k=1}^{r} \sigma_k \mathbf{X}_k, \tag{9}$$

where columns $\vec{u}_k$ of the $K \times K$-dimensional matrix **U**, also called *left-singular vectors*, and the columns $\vec{v}_k$ of the $L \times L$-dimensional matrix **V** (i.e. rows of $\mathbf{V}^T$), also called *right-singular vectors*, span the elementary (rank-1) matrices $\mathbf{X}_k = \vec{u}_k \vec{v}_k^T \equiv \vec{u}_k \otimes \vec{v}_k$, which can be thought of as "sub phase-spaces" of phase space **X**. The $K \times L$-dimensional matrix Σ contains only diagonal elements which are the ordered singular values $\sigma_1 \geq \sigma_2 \geq \ldots \geq \sigma_r$. The square of the singular value $\lambda_k = \sigma_k^2$ is the *partial variance* corresponding to the particular "sub phase-space" $\mathbf{X}_k$. The sum $\lambda_{\text{tot}} = \sum_{k=1}^{r} \lambda_k$ gives the total variance of the phase space **X**, and a so-called scree diagram can be constructed to visually represent the ordered fractional partial variances $\lambda_k/\lambda_{\text{tot}}$. Here, r is the rank of matrix **X**, i.e. the number of independent columns or rows of **X**, with $r \leq \min(K, L)$, such that the number of "sub phase-spaces" can be controlled with parameter L. By inverse transformation each elementary matrix $\mathbf{X}_k$ is converted to time series component $g_k(n)$, such that an exact decomposition of the original time series is obtained, see Eq (7). In general, the individual elementary matrices $\mathbf{X}_k$ are not of Hankel type. Therefore, the nth element of time-series component $g_k(n)$ is calculated by taking the average over the nth ascending diagonal of $\mathbf{X}_k$, a process called *diagonal averaging*. Because of the lack of Hankel symmetry of the elementary matrices $\mathbf{X}_k$, the various time-series components $g_k(n)$, with $k = 1, \ldots, r$ are not necessarily uncorrelated; graphical tools such as the scree diagram and the w—correlation matrix allow to estimate the degree up to which different time-series components $g_k(n)$ are uncorrelated (see Figs 8 and 9 in S1 File).

Supporting information

S1 File. Technical details on calculations. Calculations with Fourier spectral analysis, cosinor, intradaily variability (IV), singular spectrum analysis (SSA), kurtosis and linear trend, and detrended fluctuation analysis (DFA).
(PDF)

Acknowledgments

R.F. acknowledges fruitful discussions with M. Corsi-Cabrera.

Author Contributions

Conceptualization: Ruben Fossion, Jason Ellis, Maia Angelova.

Data curation: Ruben Fossion, Juan C. Toledo-Roy, Jason Ellis.

Formal analysis: Ruben Fossion, Juan C. Toledo-Roy, Jason Ellis, Maia Angelova.

Funding acquisition: Ruben Fossion, Maia Angelova.

Investigation: Ruben Fossion, Ana Leonor Rivera, Juan C. Toledo-Roy.

Methodology: Ruben Fossion, Ana Leonor Rivera, Juan C. Toledo-Roy.

Project administration: Ruben Fossion, Maia Angelova.

Resources: Ruben Fossion.

Software: Ruben Fossion, Ana Leonor Rivera, Juan C. Toledo-Roy.

Supervision: Ruben Fossion, Maia Angelova.

Validation: Ruben Fossion, Jason Ellis.

Visualization: Ruben Fossion.

Writing – original draft: Ruben Fossion, Maia Angelova.

Writing – review & editing: Jason Ellis, Maia Angelova.

References

1. Halberg F, Cornélissen G, Katinas G, Syutkina EV, Sothern RB, Zaslavskaya R, et al. Transdisciplinary unifying implications of circadian findings in the 1950s. J Circ Rhythms 2003; 1: 2.
2. Witting W, Kwa IH, Eikelenboom P, Mirmiran M, Swaab DF Alterations in the circadian rest-activity rhythm in aging and Alzheimer's disease Biol Psych. 1990; 27: 563–572. https://doi.org/10.1016/0006-3223(90)90523-5
3. Zee PC, Attarian H, Videnovic A Circadian Rhythm Abnormalities Continuum 2013; 19(1): 132–147. https://doi.org/10.1212/01.CON.0000427209.21177.aa PMID: 23385698
4. Gorka Z, Moryl E, Papp M. Effect of chronic mild stress on circadian rhythms in the locomotor activity in rats. Pharmacol Biochem Be. 1996; 54(1): 229–234. https://doi.org/10.1016/0091-3057(95)02173-6
5. Endo Y, Shiraki K. Behavior and body temperature in rats following chronic foot shock or psychological stress exposure. Physiol Behav. 2000; 71(3): 263–268. https://doi.org/10.1016/S0031-9384(00)00339-5 PMID: 11150557
6. Tornatzky W, Miczek KA. Long-term impairment of autonomic circadian rhythms after brief intermittent social stress. Physiol Behav. 1993; 53(5): 983–993. https://doi.org/10.1016/0031-9384(93)90278-N PMID: 8511216
7. Harper DG, Tornatzky W, Miczek KA. Stress induced disorganization of circadian and ultradian rhythms: comparisons of effects of surgery and social stress. Physiol Behav. 1996; 59(3): 409–419. https://doi.org/10.1016/0031-9384(95)02012-8 PMID: 8700940
8. Frank E, Benabou M, Bentzley B, Bianchi M, Goldstein T, Konopka G, et al. Influencing circadian and sleep—wake regulation for prevention and intervention in mood and anxiety disorders: What makes a good homeostat?. Ann NY Acad Sci. 2014; 1334(1): 1–25. https://doi.org/10.1111/nyas.12600 PMID: 25532787
9. McClung CA. Circadian rhythms and mood regulation: insights from pre-clinical models. Eur Neuropsychopharm. 2011; 21: S683–S693. https://doi.org/10.1016/j.euroneuro.2011.07.008
10. Hall M, Vasko R, Buysse D, Ombao H, Chen Q, Cashmere JD, et al. Acute stress affects heart rate variability during sleep. Psychosom Med. 2004; 66(1): 56–62. https://doi.org/10.1097/01.PSY.0000106884.58744.09 PMID: 14747638
11. Hall M, Thayer JF, Germain A, Moul D, Vasko R, Puhl M, et al. Psychological stress is associated with heightened physiological arousal during NREM sleep in primary insomnia. Behav Sleep Med. 2007; 5(3): 178–193. https://doi.org/10.1080/15402000701263221 PMID: 17680730

12. Morris M, Lack L, Dawson D. Sleep-onset insomniacs have delayed temperature rhythms. Sleep. 1990; 13(1): 1–14. https://doi.org/10.1093/sleep/13.1.1 PMID: 2305166

13. Lack L, Wright H. The effect of evening bright light in delaying circadian rhythms and lengthening the sleep of early morning awakening insomniacs. Sleep. 1993; 16: 436–443 https://doi.org/10.1093/sleep/16.5.436 PMID: 8378685

14. Kripke DF, Rex KM, Ancoli-Israel S, Nievergelt CM, Klimecki W, Kelsoe JR. Delayed sleep phase cases and controls. J Circ Rhythms. 2008; 6: 6.

15. Bonmati-Carrion MA, Middleton B, Revell V, Skene DJ, Rol MA, Madrid JA (2014). Circadian phase asessment by ambulatory monitoring in humans: Correlation with dim light melatonin onset. Chronobiol. Int. 2013; 31(1): 37–51. https://doi.org/10.3109/07420528.2013.820740

16. Littner M, Hirshkowitz M, Kramer M, Kapen S, Anderson WM, Bailey D, et al. Practice parameters for using polysomnography to evaluate insomnia: an update. Sleep. 2003; 26(6):754–60. PMID: 14572131

17. Morgenthaler T, Kramer M, Alessi C, Friedman L, Boehlecke B, Brown T, et al. Practice parameters for the psychological and behavioral treatment of insomnia: an update. Sleep. 2006; 29(11): 1415–1419. PMID: 17162987

18. Natale V, Plazzi G, Martoni M. Actigraphy in the assessment of insomnia: a quantitative approach. Sleep. 2009; 32(6): 767–771. https://doi.org/10.1093/sleep/32.6.767 PMID: 19544753

19. Lichstein KL, Stone KC, Donaldson J, Nau SD, Soeffing JP, Murray D, et al. Actigraphy validation with insomnia. Sleep. 2006; 29(2): 232–239. PMID: 16494091

20. Sadeh A. The role and validity of actigraphy in sleep medicine: an update. Sleep Med Rev. 2011; 15(4), 259–267. https://doi.org/10.1016/j.smrv.2010.10.001 PMID: 21237680

21. Halberg F, Tong YL, Johnson EA Circadian system phase—An aspect of temporal morphology: Procedures and illustrative examples Springer Berlin Heidelberg: 1967, pp.20–48.

22. Cornelissen G. Cosinor-based rhythmometry Theo Biol Med Model 2014; 11: 16. https://doi.org/10.1186/1742-4682-11-16

23. Krishnan B, Levine JD, Lynch MKS, Dowse HB, Funes P, Hall JC, et al. A new role for cryptochrome in a Drosophila circadian oscillator. Nature. 2001 May; 411: 313–317. https://doi.org/10.1038/35077094 PMID: 11357134

24. Levine JD, Funes P, Dowse HB, Hall JC Signal analysis of behavioral and molecular cycles. BMC Neurosci. 2002; 3:1. https://doi.org/10.1186/1471-2202-3-1 PMID: 11825337

25. Yu W, Hardin PE Use of firefly luciferase activity assays to monitor circadian molecular rhythms in vivo and in vitro In: Methods in molecular biology, Vol.362, Circadian rhythms: Methods and protocols, Ed. Rosato E New Jersey: Humana Press.; 2007.

26. Dowse HB Analyses for physiological and behavioral rhythmicity. In: Methods in Enzymology, Vol.454. Elsevier.; 2009.

27. Meeker K, Harang R, Webb AB, Welsh DK, Doyle FJ III, Bonnet G, et al. Wavelet measurement suggests cause of period instability in mammalian circadian neurons. J Biol Rhythms. 2011; 26(4): 353–362. https://doi.org/10.1177/0748730411409863 PMID: 21775294

28. Leise TL, Harrington ME. Wavelet-based time series analysis of circadian rhythms. J Biol Rhythms. 2011; 26(5): 454–463. https://doi.org/10.1177/0748730411416330 PMID: 21921299

29. Gonçalves BSB, Adamowicz T, Mazzilli Louzada F, Moreno CR, Fontenele Araujo J A fresh look at the use of nonparametric analysis in actimetry Sleep Med Rev. 2015 Apr; 20:84–91. https://doi.org/10.1016/j.smrv.2014.06.002 PMID: 25065908

30. Gonçalves BSB, Adamowicz T, Mazzilli Louzada F, Moreno CR, Fontenele Araujo J Nonparametric methods in actigraphy: An update Sleep Sci 2014 Sept; 158–164. https://doi.org/10.1016/j.slsci.2014.09.013 PMID: 26483921

31. Refinetti R, Cornelissen G, Halberg F Procedures for numerical analysis of circadian rhythms Biol Rhythm Res 2007; 38(4): 275–325. https://doi.org/10.1080/09291010600903692 PMID: 23710111

32. Iyengar N, Peng C-K, Morin R, Goldberger AL, Lipsitz LA Age-related alterations in the fractal scaling of cardiac interbeat interval dynamics Am J Physiol. 1996; 271: R1078–R1084. PMID: 8898003

33. Peng C-K, Havlin S, Hausdorff M, Mietus JE, Stanley HE, Goldberger AL. Non-random fluctuations and multi-scale dynamics regulation of human activity. Phys A. 2004; 337: 307–318. https://doi.org/10.1016/j.physa.2004.01.042

34. Hu K, Ivanov PCh, Chen Z, Hilton MF, Stanley HE, Shea SA. Non-random fluctuations and multi-scale dynamics regulation of human activity. P Natl Acad Sci USA. 2009; 106(8): 2490–2494.

35. Ivanov PCh, Hu K, Hilton MF, Shea SA, Stanley HE. Endogenous circadian rhythm in human motor activity uncoupled from circadian influences on cardiac dynamics. P Natl Acad Sci USA. 2007; 104(52): 20702–20707. https://doi.org/10.1073/pnas.0709957104

36. Hu K, Van Someren EJW, Shea SA, Scheer FAJL. Reduction of scale invariance of activity fluctuations with aging and Alzheimer's disease: Involvement of the circadian pacemaker. P Natl Acad Sci USA. 2009; 106(8): 2490–2494. https://doi.org/10.1073/pnas.0806087106
37. Elsner JB, Tsonis AA Singular Spectrum Analysis: A new tool in time series analysis Springer Science +Business Media, LLC: New York.
38. Golyandina N, Nekrutkin V, Zhigljavsky AA Analysis of time series structure: SSA and related techniques Chapman & Hall/CRC: Boca Raton, London, New York, Washington D.C.
39. Golyandina N, Zhigljavsky A Singular spectrum analysis for time series Springer Briefs in statistics: Heidelberg, New York, Dordrecht, London
40. Hassani H Singular Spectrum Analysis: Methodology and comparisons J Data Sci 2007; 5: 239–257.
41. Ghil M, Allen MR, Dettinger MD, Ide K, Kondrashov D, Mann ME, et al. Advanced spectral methods for climatic time series Rev Geophys. 2002; 40: 1. https://doi.org/10.1029/2000RG000092
42. Huang NE, Shen Z, Long SR, Wu MC, Shih HH, Zheng Q, et al. The empirical mode decomposition and the Hilbert spectrum for nonlinear and non-stationary time series analysis. P Roy Soc A-Math Phys. 1998; 454(1971): 903–995. https://doi.org/10.1098/rspa.1998.0193
43. Huang NE, Wu MC, Man-Li C, Long SR, Shen SSP, Qu W, et al. A confidence limit for the empirical mode decomposition and Hilbert spectral analysis. P Roy Soc A-Math Phys. 2003; 459(2037): 2317–2345. https://doi.org/10.1098/rspa.2003.1123
44. Iatsenko D, McClintok PVE, Stefanofska A. Nonlinear mode decomposition: A noise-robust, adaptive decomposition method Phys Rev E. 2015; 92: 032916. https://doi.org/10.1103/PhysRevE.92.032916
45. Iatsenko D. Nonlinear mode decomposition: Theory and applications Cham Heidelberg New York Dordrecht London: Springer.; 2015.
46. Yamazaki S, Kerbeshian MC, Hocker CG, Block GD, Menaker M. Rhythmic properties of the hamster suprachiasmatic nucleus in vivo J Neurosci. 1998; 18(24) 10709–10723. PMID: 9852606
47. http://www.msandri.it/soft.html
48. http://research.atmos.ucla.edu/tcd///ssa/matlab/
49. https://cran.r-project.org/web/packages/Rssa/index.html
50. https://github.com/travisszucs/ssa-research
51. http://mathematica.stackexchange.com/questions/28724/hilbert-huang-transform-package
52. http://perso.ens-lyon.fr/patrick.flandrin/software2.html
53. https://cran.rstudio.com/web/packages/EMD/index.html
54. https://github.com/wmayner/pyemd
55. http://www.physics.lancs.ac.uk/research/nbmphysics/diats/
56. Holloway PM, Angelova M, Lombardo S, St Clair Gibson A, Lee David, Ellis J. Complexity analysis of sleep and alterations with insomnia based on non-invasive techniques. Interface 2014 Feb; 11: 20131112. https://doi.org/10.1098/rsif.2013.1112 PMID: 24501273
57. Kleitman N. Sleep and wakefulness. Chicago: University Press; 1939.
58. Lavie P. Ultradian cycles in sleep propensity: Or, Kleitman's BRAC revisited In: Ultradian rhythms in life processes: An inquiry into fundamental principles of chronobiology and psychobiology, Eds. Lloyd D and Rossi EL. London: Springer-Verlag; 1992.
59. Okudaira N, Kripke DF, Webster JB. No basic rest-activity cycle in head, wrist or ankle Physiol Behav. 1984; 32: 843–845. PMID: 6494289
60. Lavie P. Ultradian rhythms in arousal—The problem of masking. Chronobiol Int. 1989; 6(1): 21–28. https://doi.org/10.3109/07420528909059139 PMID: 2706701
61. Sing HC. Human biological rhythms: The search for ultradians in movement activity behaviour In: Ultradian rhythms in life processes: An inquiry into fundamental principles of chronobiology and psychobiology, Eds. Lloyd D and Rossi EL. London: Springer-Verlag; 1992.
62. Grau C, Escera C, Cilveti R, García M, Mojón A, Fernández JR et al. Ultradian rhythms in gross motor activity of adult humans. Physiol Behav. 1995; 57(3): 411–419. https://doi.org/10.1016/0031-9384(94)00274-9 PMID: 7753876
63. Niggemyer KA, Begley A, Monk T, Buysse DJ Circadian and homeostatic modulation of sleep in older adults during a 90-minute day study. Sleep. 2004; 27(8): 1535–1541. https://doi.org/10.1093/sleep/27.8.1535 PMID: 15683145
64. Hayashi M, Sato K, Hori T Ultradian rhythms in task performance, self-evaluation, and EEG activity. Perceptual and motor skills. 1994; 79(2): 791–800. https://doi.org/10.2466/pms.1994.79.2.791 PMID: 7870505

65. Krampe RT, Ericsson KA Deliberate practice and elite musical performance. Chapter 4 in: The practice of performance: Studies in musical interpretation, Ed. John Rink, Cambridge Univ. Press, Cambridge, 1995, pp. 84–102.

66. Ericsson KA (2015) Acquisition and maintenance of medical expertise: A perspective from the expert-performance approach with deliberate practice. Acad. Med. 2015; 90(11): 1471–1486.

67. Goldberger AL, Amaral LAN, Hausdorff JM, Ivanov PCh, Peng CK, Stanley HE. Fractal dynamics in physiology: Alterations with disease and aging. P Natl Acad Sci USA. 2002; 99(1): 2466–2472. https://doi.org/10.1073/pnas.012579499

68. Fossion R. A time-series approach to dynamical systems from classical and quantum worlds. AIP Conf Proc. 2014; 1575: 89–110. https://doi.org/10.1063/1.4861700
